# Generation of a Matrix Gla (*Mgp*) floxed mouse, followed by conditional knockout, uncovers a new *Mgp* function in the eye

**DOI:** 10.1038/s41598-020-75031-7

**Published:** 2020-10-29

**Authors:** Teresa Borrás, Dale O. Cowley, Priyadarsini Asokan, Kumar Pandya

**Affiliations:** 1 Department of Ophthalmology, University of North Carolina School of Medicine, 4109C Neuroscience Research Building CB 7041, 115 Mason Farm Road, Chapel Hill, NC 27599-7041 USA; 2 Animal Models Core, University of North Carolina, Chapel Hill, NC USA

**Keywords:** Glaucoma, Calcification, Genetic transduction, Animal breeding, Bone, Experimental models of disease, Cancer metabolism, Animal disease models, Cardiovascular models, Genetic models, Mouse, Molecular medicine

## Abstract

The ability to ablate a gene in a given tissue by generating a conditional knockout (cKO) is crucial for determining its function in the targeted tissue. Such tissue-specific ablation is even more critical when the gene’s conventional knockout (KO) is lethal, which precludes studying the consequences of its deletion in other tissues. Therefore, here we describe a successful strategy that generated a Matrix Gla floxed mouse (*Mgp.floxed*) by the CRISPR/Cas9 system, that subsequently allowed the generation of cKOs by local viral delivery of the Cre-recombinase enzyme. MGP is a well-established inhibitor of calcification gene, highly expressed in arteries’ smooth muscle cells and chondrocytes. MGP is also one of the most abundant genes in the trabecular meshwork, the eye tissue responsible for maintenance of intraocular pressure (IOP) and development of Glaucoma. Our strategy entailed one-step injection of two gRNAs, Cas9 protein and a long-single-stranded-circular DNA donor vector (lsscDNA, 6.7 kb) containing two loxP sites *in cis* and 900–700 bp 5′/3′ homology arms. Ocular intracameral injection of *Mgp.floxed* mice with a Cre-adenovirus, led to an *Mgp.*TMcKO mouse which developed elevated IOP. Our study discovered a new role for the *Mgp* gene as a keeper of physiological IOP in the eye.

## Introduction

To understand the function of a particular gene and its effect on the pathophysiology of a disease, investigators have relied on the generation of animal models where the target gene has been modified or ablated^1,2^. While the mouse’s physiology does not fully mimic that of human, the generation of whole-body gene KOs and of tissue conditional knockouts (cKOs) in this species has led to countless discoveries^3,4^. The traditional, more cumbersome methods of generating mouse models by gene targeting embryonic stem cells^5,6^ have been mostly replaced by highly precise gene editing technology that inserts loxP sites flanking an essential DNA fragment of the target gene and make it available to a Cre-recombinase for its targeted mutation^7,8^. This gene editing system, termed clustered regularly interspaced short palindromic repeats (CRISPR)/CRISPR associated protein (Cas9) further allows the precise insertion of exogenous DNA sequences. By directly injecting a combination of the Cas9 endonuclease, guide-RNAs (gRNA) complementary to the target site, and the desired editing donor DNA in zygotes, the mouse genome can get edited at the precise target site^9,10^. The Cas9/gRNA complex binds to the target region in the genome which is complementary to the target gRNA and is followed by a protospacer associated sequence (PAM) of NGG. The Cas9/gRNA complex then introduces a double-stranded DNA break (DSB) into the genome^11–13^. The cellular repair of a DSB can utilize a homology dependent repair pathway in which a donor DNA with exogeneous sequences flanked by homology regions corresponding to the DNA flanking the DSB, can be integrated at the DSB site. In this study, we report the generation of an edited floxed Matrix Gla (*Mgp*) mouse, containing two loxP sites located *in cis* 2086 bp apart. The edited mouse was thoroughly validated in vitro and in vivo for its ability to functionally produce a cKO with a local *Mgp* trabecular meshwork ablation.

Matrix Gla (MGP) is a Vitamin-K-dependent protein shown to be an inhibitor of calcification in the extracellular matrix (ECM). MGP is translated as a 104 amino-acid (aa) protein which undergoes post-translational modifications by a Vitamin-K-dependent γ-carboxylase (GGCX) (five glutamic acid residues to Gla) and a Golgi-casein kinase (phosphorylation of 3 serine residues) to produce the active protein^14–17^. It is well-established that the active, secreted, 84 aa mature protein, acts as a potent local inhibitor of calcification and protects soft tissue from hardening and stiffness^18–23^. MGP was initially discovered in bone^24^ and cartilage^25^ and later shown to be highly expressed in vascular smooth muscle cells (VSMC) of the tunica media of arteries, where it played a critical role to prevent vessel calcification^18^. Mutations in the *MGP* gene have been associated with Keutel syndrome, an autosomal recessive rare disease first described in 1972^20,26,27^. To date only 36 confirmed cases globally have been reported. Patients with this disease develop abnormal calcification in cartilage, lungs and vascular system. The severity of the disease depends on the extent of pulmonary involvement, and reports indicate that those patients die at a young age. Although very few ophthalmological exams have been described, the case of one 6-year old experiencing sudden loss of vision in both eyes and bilateral optic nerve atrophy has been reported^27^.

The initial MGP expression profile was later expanded to several other systemic tissues, such as the kidney, lung and eye^28–31^ and more recently, its expression has been found to be altered in tumors associated with several types of cancers^32^. MGP has also been implicated in the extracellular homeostatic calcium regulation of sperm maturation^33^. Earlier studies from our laboratory identified *MGP* as one of the most abundant non-housekeeping genes of the human trabecular meshwork of the eye^29,34–37^, which is the tissue responsible for the maintenance of physiological intraocular pressure (IOP). In the mouse, our recently generated transgenic *Mgp-*Cre.KI line, crossed with floxed reporters, confirmed the abundance and specific localization of *Mgp* to the mouse’s eye trabecular meshwork region, and revealed a second expression site in the peripapillary sclera and retinal vasculature, including capillaries and pericytes^38,39^. These eye tissues are all known to be relevant in the development of glaucoma. A recent clinical longitudinal study showed that high levels of circulating, inactive MGP (dephospho-uncarboxylated) could be predictive of narrowing of the retinal microvessels^40^, a trait that is indicative of glaucomatous vascular signs^41,42^. In the human trabecular meshwork, the MGP protein is present in its active conformation, and the levels of the activating enzyme γ-carboxylase are very high^36^. In addition, MGP is a mechanical responsive gene and its transcription in the trabecular meshwork is altered by elevated IOP, stretch, and by IOP inducing agents^34,35,37,43,44^. As well, MGP transcription is reduced in glaucomatous patients^45^. All these characteristics suggested that *MGP* is a very relevant gene to eye function. The increasing information about the presence of MGP in different tissues, and its association with different diseases opened the question as to whether MGP exerts its central calcium binding/inhibition of calcification function through different pathways, which could affect each tissue in a different manner. We reasoned that the potential of ablating the *Mgp* gene, locally and specifically, in any given tissue of a living animal would be essential to understand its functional calcium-related cascade. In this study, we thus embarked on the process of generating a floxed *Mgp* mouse to be able to create local cKO by local viral delivery of the Cre-recombinase. Because of our primary interest in trabecular meshwork and glaucoma, we used the newly generated *Mgp.floxed* mouse to assess Mgp’s role in the main function of this tissue, the maintenance of physiological IOP.

Glaucoma is a complex optic neuropathy that results in irreversible blindness^46^. In all its forms, glaucoma is the leading cause of irreversible blindness worldwide^47^. Although some cases exist where the presence of the disease occurs without elevated IOP^48^, it is well-established that elevated IOP is the major risk factor for the development of glaucoma^49^. Currently, there is no cure for glaucoma and IOP reduction remains the only treatment strategy for all forms of the disease, including normal tension glaucoma^49^. Physiological and/or elevated IOP is determined by the resistance offered to aqueous humor flow by the trabecular meshwork outflow pathway^50^, a spongiform tissue located at the corner of the iris and the cornea. Thus, the discovery of the function of the genes highly expressed in trabecular meshwork would be essential for understanding IOP regulatory mechanisms, and for the development of therapies aiming to reduce IOP and prevent glaucoma-caused blindness.

In view of these observations, we hypothesized that the *MGP* gene might play a key role in the physiological function of the trabecular meshwork/outflow pathway. To test this hypothesis, in this study, we generated an *Mgp.floxed* mouse and assessed the consequences of ablating the *Mgp* gene by local viral delivery of the Cre-recombinase, in the regulation of IOP.

## Results

### CRISPR/Cas9-mediated gene editing for the generation of an *Mgp.floxed* mouse

**Figure 1:**
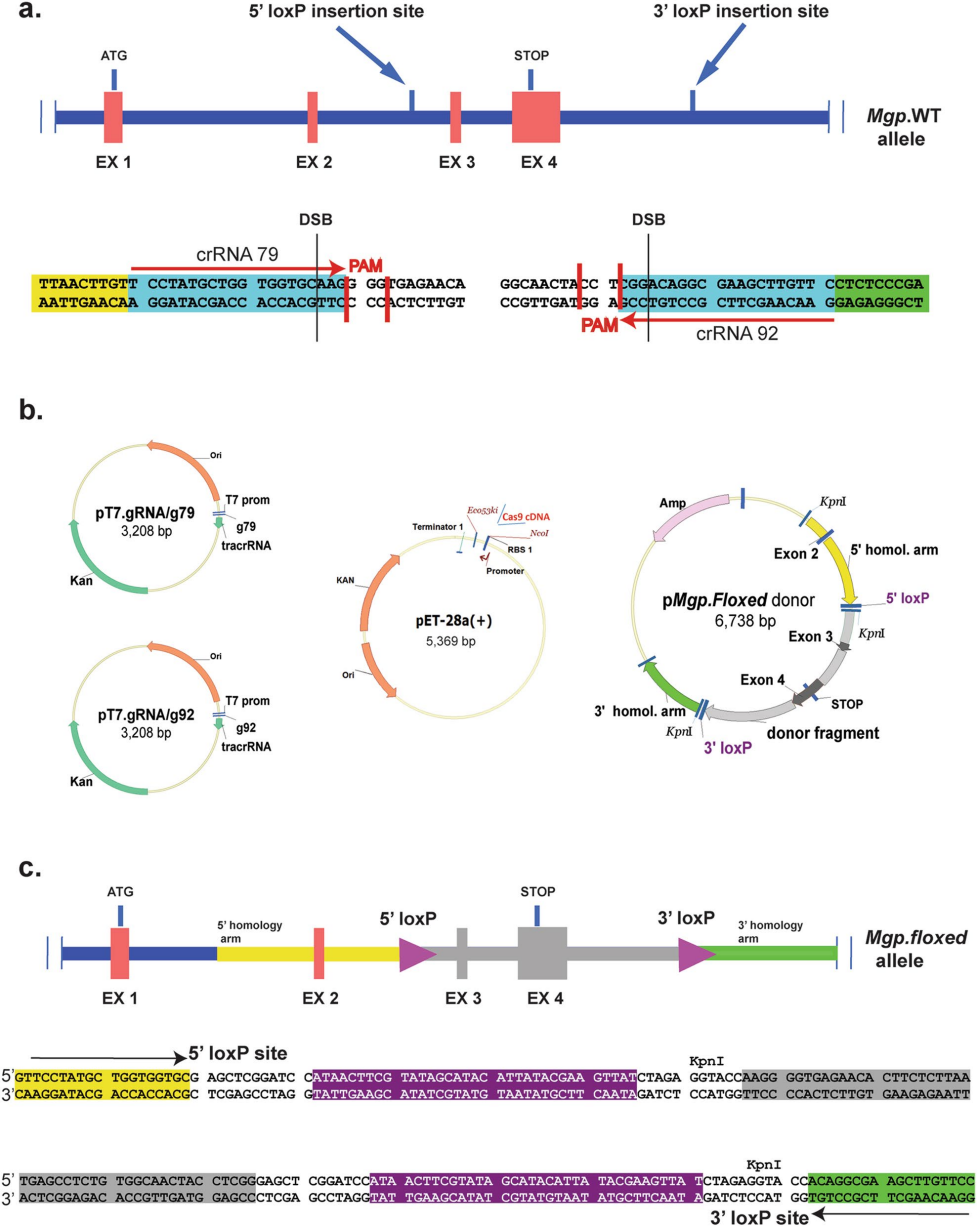
Schematic diagram of the overall gene editing strategy for the generation of an *Mgp.floxed* mouse. (**a**) Diagram of the *Mgp.*WT mouse allele and identification of the 5′ and 3′ loxP insertions sites; detailed sequences of the insertion sites showing the orientation and selection of the crRNA sequences, the Protospacer Adjacent Motives (PAM) and putative DNA double-stranded breaks (DSB) locations. (**b**) Key plasmids constructed for the generation of the two gRNAs, Cas9 protein, and donor vector p*Mgp.Floxed.* (**c**) Diagram of the *Mgp.floxed* allele showing the DNA elements included in the p*Mgp.Floxed* donor vector; yellow: 5′ homology arm; grey: DNA targeted for recombination; green: 3′ homology arm; purple arrowheads: loxP sites. Detailed sequences surrounding the 5′ (*top*) and 3′ (*bottom*) loxP sites. Arrows: sequences of the 5′ and 3′ crRNAs (crRNA79 and crRNA92).

The *MGP* gene is highly expressed in the trabecular meshwork of at least human^29,34–37^, porcine^44^ and mice^38,39^. Because the *Mgp* KO^18^ is lethal and because of the need to address the potential relevance of MGP in trabecular meshwork physiology, we set up to generate an *Mgp.floxed* mouse that would then allow the creation of a trabecular meshwork cKO (*Mgp.*TMcKO). Our strategy made use of the CRISPR/CAS9-mediated gene editing technology strategies to allow insertion of two loxP sequences in the same *Mgp* allele (*in cis*). A diagram with the overall design of the needed elements for this gene editing is shown in Fig. 1. We identified insertion sites in the mouse genome, generated optimized gRNAs and Cas9 expression plasmids and constructed a donor DNA vector with two restriction sites-flanked loxP sequences (Fig. 1).

The *Mgp* gene contains four exons with initiation of translation at 66 nt in of exon 1 and termination at 145 nt in of exon 4 (MGI: 96976; GeneID: 17313). Our *Mgp.floxed* mouse design to allow the subsequent generation of an *Mgp* cKO, entailed deletion of a region containing exons 3 and 4, which encode 70% of the protein’s C-terminus (aa 12 to 104 Mgp) and will produce a small truncated inactive protein. This truncated protein, encoded by remaining exons 1 and 2, would be predicted to be 31 aa, from which 19 aa comprise the signal peptide. Thus, the secreted product would be approximately 1.5 kDa. The exon 4 deletion comprises also the mRNA’s 3′ UTR and would most likely affect mRNA levels. In addition, this DNA region includes critical functional binding sites^51^ whose elimination by themselves will render an inactive Mgp protein. To find loxP insertion sites, intron 2 and the 3′ proximal genomic region of *Mgp* were searched using https://cripsr.mit.edu/ and https://zlab.bio/guide-design-resources databases to identify potential Cas9 guides RNA with low off-target mutagenesis. We identified sites at 374 nt upstream of exon 3 in the sense strand for the 5′ loxP site, and 904 nt downstream of exon 4 in the antisense strand. Predictive algorithms yielded two CRISPR RNA (crRNA) sequences with numerical off-target sites scores of 77 and 79 for the 5′ loxP insertion region, and two sequences with scores of 65 and 92, for the 3′ loxP insertion region. The four crRNA candidates were fused to the Cas9 binding scaffold trans-activating CRISPR RNA (tracrRNA) to generate single guide-RNAs (gRNAs).

**Figure 2:**
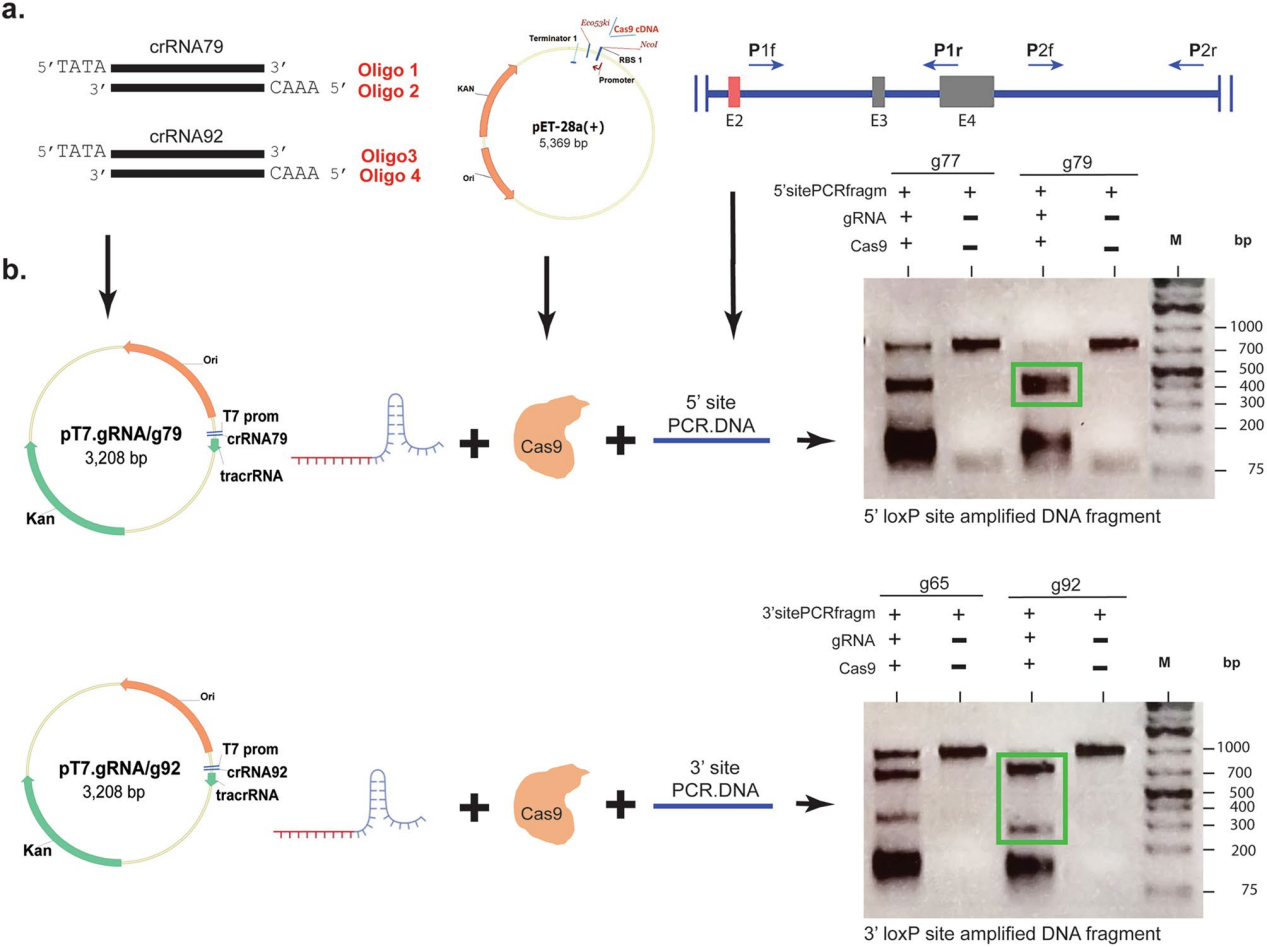
Assay of DSB cutting efficiency of the four generated gRNAs in vitro. (**a**) Diagram of the elements used in the assay. Oligonucleotides annealed for the generation of crRNAs used in the transcription vectors (left), pET-28a(+) plasmid used to produce the human codon optimized Cas9 protein (center) and mouse DNA diagram showing location of the primers (arrows) used to amplify the DNA fragments used in the assay (right). (**b**) Incubation of in vitro transcription from gRNA plasmids, Cas9 and PCR-generated DNA (left); electrophoretic separation of the resulting DNA fragments in a 2% agarose/TAE gel stained with ethidium bromide (right). M: 1 kb Plus DNA Ladder (GeneRuler, ThermoFisher). For the 5′ site, the P1f/P1r 785 bp amplified fragment incubated with the gRNAs resulted in a doublet band corresponding to the 425 bp and 358 bp fragments; the gRNAg79 was more efficient than gRNAg77. For the 3′ site, the P2f/P2r 985 bp amplified fragment incubated with the gRNAs resulted in 837 bp and 248 bp fragments; the gRNA92 was more efficient than gRNAg65. Size of the fragments correspond to the predicted DSB (relevant gel bands encased in green boxes for easier visualization). Lower MW bands correspond to primer dimers. Incubations controls without the gRNAs and Cas9 protein were negative.

For the generation of each gRNA, two complementary 20 nt oligonucleotides per site were synthetized containing an addition of 4 overhanging nt (5′ TATA to the sense strands and 5′ AAAC to the antisense strands to allow cloning) (Table 1 for the two selected crRNAs) (Fig. 2). Complementary oligonucleotides were annealed and cloned into an in-house custom-made T7 expression plasmid (pT7.gRNA g79/92) between two BsaI proximal cutters located downstream of the T7 promoter and upstream of the 77 bp tracrRNA (Cas9 binding site). In vitro transcription with T7 RNA polymerase produced single 97 bp gRNAs which were purified on RNeasy columns. Cas9 protein cDNA^13^ was cloned into prokaryotic expression plasmid pET-28a(+), expressed in bacteria and purified by Ni–NTA agarose. DSB cutting efficiency of the four transcribed gRNAs was assayed in vitro with the Cas9 protein and with C57BL/6J (B6) mouse genome DNA fragments amplified with primers P1f/P1r and P2f/P2r (Table 1). The primers surround the 5′ and 3′ potential loxP insertions sites and produced 785 bp and 985 bp fragments respectively. These PCR amplicons (250 ng) were incubated with 600 ng each of the corresponding gRNA and 100 nM Cas9 at 37 °C. Gel electrophoresis analyses of resulting fragments showed that gRNAs g79 and g92 (5′ and 3′ loxP sites respectively) were the more efficient and each rendered DNA fragments whose size corresponded to the utilization of the expected DSB close to 100% efficiency (Fig. 2).

**Table 1:**
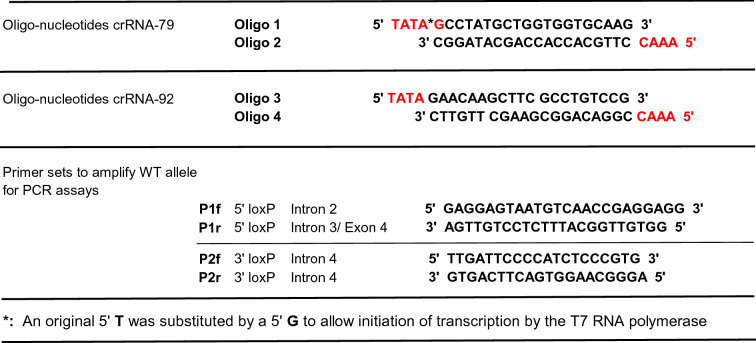
Sequence of cRNAs oligos and of primers used on their in vitro functional assay.

*An original 5′ T was substituted by a 5′ G to allow initiation of transcription by the T7 RNA polymerase.

**Figure 3:**
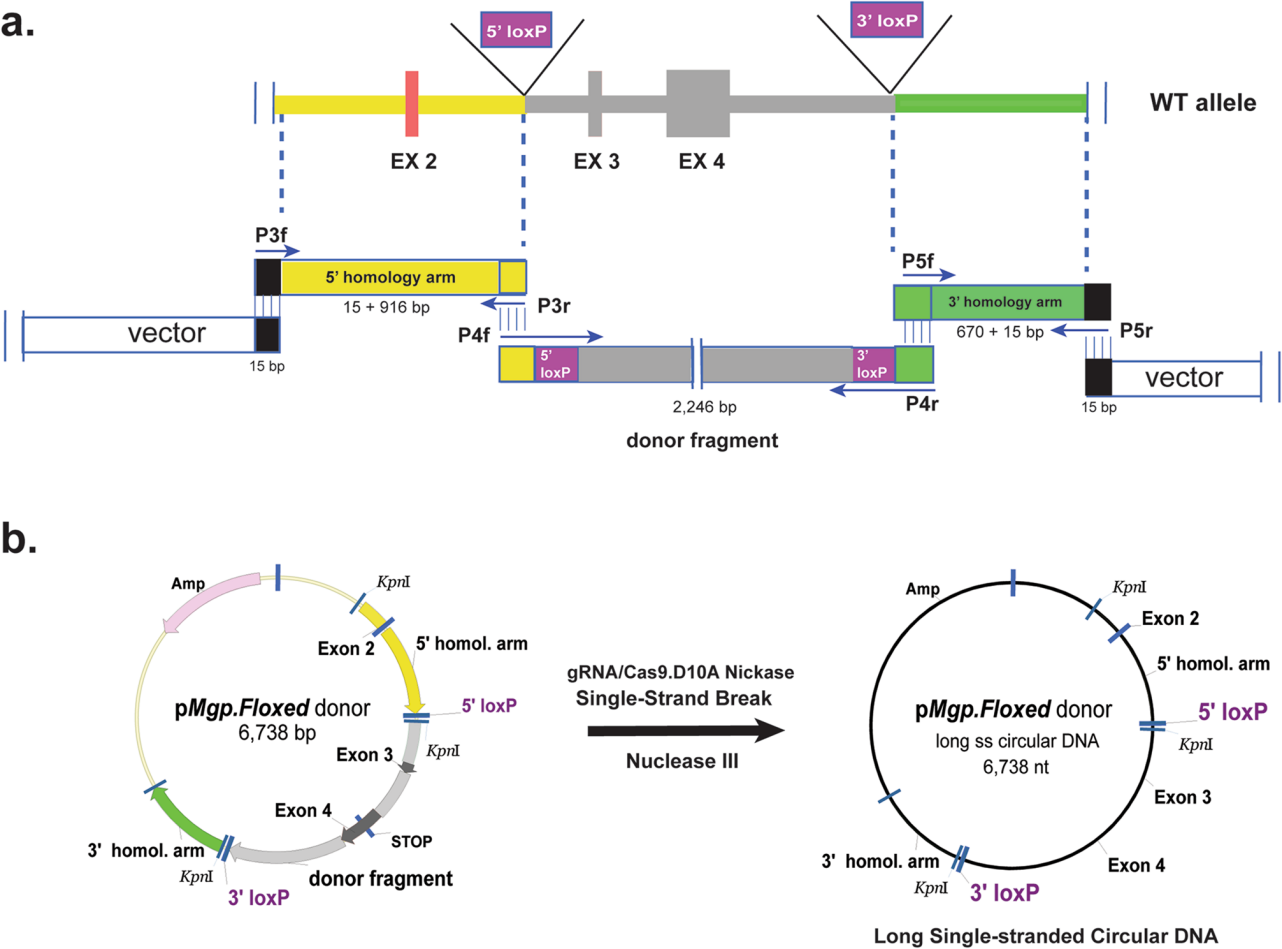
Generation of the long-single-stranded-circular (lssc) donor vector (p*Mgp.Floxed*) containing 5′ and 3′ loxP sequences *in cis*. (**a**) Strategy of the In-Fusion cloning: (*top*) diagram of the B6 genomic region depicting the location of the three DNA elements along the *Mgp.*WT allele, and the designed insertion sites of the loxP sequences; (*bottom*) each of the DNA elements flanked by 15 bp sequences overlapping the previous and next DNA fragments (vector/fragment/fragment/fragment/vector). Arrows indicate primers used to PCR amplify each of the DNA elements with their corresponding fusion sequences. P3f/P3r (5′ arm, 916 bp product), P4f/P4r (donor fragment, 2,246 bp product) and P5r/P5r (3′ arm, 670 bp product). Primer sequences are shown in Table 2. Cloning details are described in “Methods” section. (**b**) Conversion of the 6,738 bp ds p*Mgp.Floxed* donor plasmid to the injection grade long-single-stranded-circular (lssc) molecule. To produce the lssc molecule, the ds DNA plasmid was first nicked by incubation with a gRNA (designed to target sequences 480 bp downstream of Ex4) and a mutated Cas9.D10A protein that produces ss breaks. The nicked plasmid was then treated with Nuclease III to degrade one strand from the 3′ end and purified. Yellow: 5′ homology arm; grey: edited donor fragment with loxP sequences; green: 3′ homology arm. black: pUC background vector.

Several attempts to insert two loxP sites *in cis* in the *Mgp* gene by injecting two separate donor DNAs failed to generate a sole *Mgp.floxed* mouse allele. Four microinjection attempts were made with single-stranded oligomers, and 2 attempts with supercoiled donor vectors without any success (Table 1S). To overcome this problem, we designed a single donor vector containing both, the 5′ and 3′ loxP sites *in cis*. The vector, p*Mgp.Floxed* donor, was generated by In-Fusion cloning of three DNA elements amplified from the B6 mouse genome and subsequent seamless insertion into a basic in-house generated pUC backbone plasmid (Fig. 3). The first element, a 916 bp 5′ homology arm (plus 15 fusion vector bp), contained sequences from 226 bp upstream to 655 bp downstream of exon 2 (primers P3f/P3r). The second element, a 2246 bp donor fragment containing both the 5′- and 3′-loxP sequences plus KpnI restriction sites, extended from 655 bp downstream of exon 2 to 1000 bp downstream of exon 4 (primers P4f/P4r). The third element, a 670 bp 3′ homology arm fragment (plus 15 fusion vector bp) extended from 1000 to 1655 bp downstream of exon 4 primers (P5f/P5r) (Table 2, Fig. 3). To allow for In-Fusion cloning, the forward primers corresponding to the sense strand of each element, contained a 15 bp sequence that complemented with 15 bp of the 5′ end of the antisense strand of the previous element (Table 2). In order to secure accurate insertion of the loxP DNA sequences plus restriction sites in the donor fragment element, these forward and reverse primers (P4f/P4r) were made to contain 160 bp oligonucleotides each (Table 2). After In-Fusion cloning, the resulting 6738 *pMgp.Floxed* donor plasmid containing a total of 3802 bp of mouse *Mgp* gene with inserted loxP sites in the noncoding region, was confirmed by sequencing (Fig. 3).

**Table 2:**
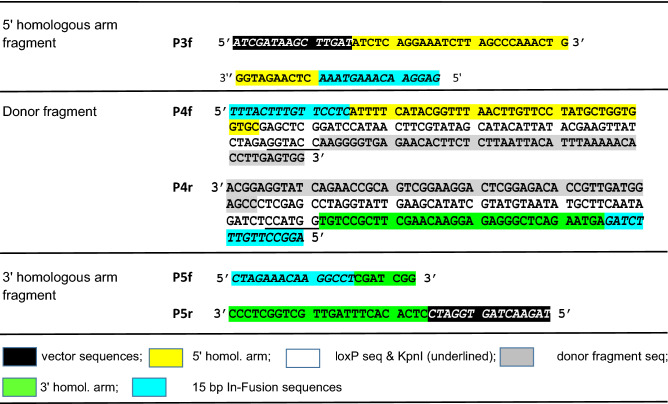
PCR primers for the In-Fusion cloning generation of the donor vector.

### Microinjection of CRISPR/Cas9 with long-single-stranded-circular DNA (lsscDNA) to generate the *Mgp-*floxed mice

Because of our failed previous attempts and, of the reported success of using single-stranded donor DNAs^10,52,53^, our plasmid donor vector was converted to a single-stranded circular DNA before injection. For this, a new gRNA with a vector-matching sequence at 480 nt downstream of exon 4 was generated (AATGGTCCCATATGTGACTA(tgg)). The double-stranded (ds) DNA donor vector was then incubated (1 h, 37 °C) with the gRNA and a mutated form of Cas9 (mutation D10A) which causes a single-stranded (ss) DNA break, rather than the DSB caused by the Cas9 WT protein^13^. The resulting nicked DNA was purified by phenol/chloroform extraction and treated with Nuclease III, which removed nucleotides from the 3′ end of nicked DNA and thus rendered a long-single-stranded-circular DNA molecule (lssc). After the treatment, the lssc DNA was purified by a Qiaquick spin column and dialyzed (Fig. 3b).

**Figure 4:**
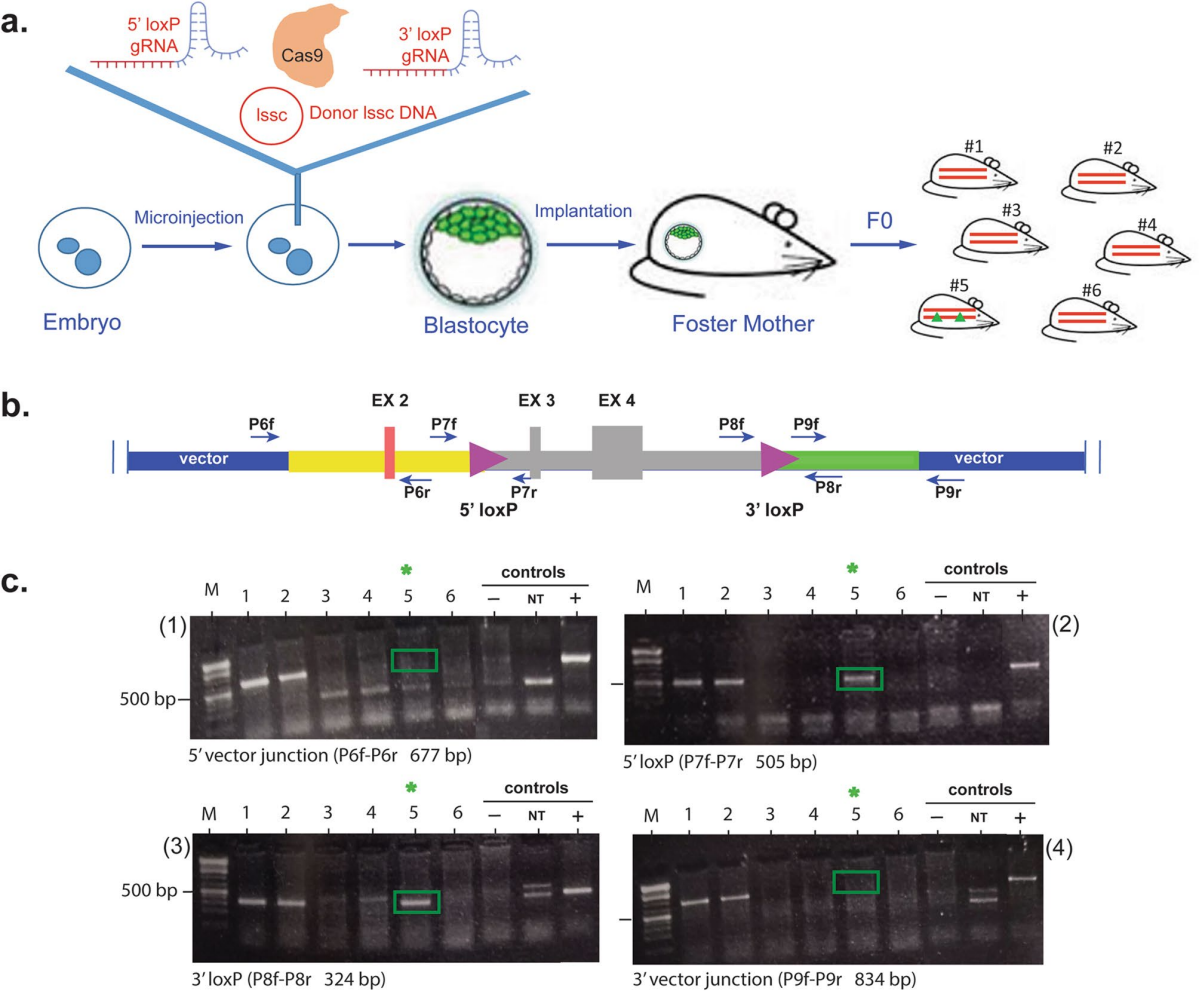
*Mgp.floxed* mouse generated by targeted embryo microinjection. (**a**) Schematic approach to generate *Mgp.floxed* alleles using a CRISPR/Cas system entailing two gRNAs (5′ and 3′) and a one long-single-stranded-circular DNA (lssc) composed of the donor/edited region flanked by two *in cis* loxP sites plus 5′ and 3′ homology arms (Fig. 3). Sixty-nine embryos were injected and of those, 21/2-cell surviving embryos were implanted in one recipient CD-1 female, which delivered six pups (#1 to #6). (**b**) Diagram of the *Mgp.floxed* allele and PCR strategy to analyze the genome of the F0 pups. Arrows indicate primers used to PCR-amplify pups DNA to identify each genotype. P6f/P6r (to identify absence of 5′ vector sequences, 677 bp product); P7f/P7r (to identify presence of 5′ loxP site, 505 bp product); P8f/P8r (to identify presence of 3′ loxP, 324 bp product) and P9f/P9r (to identify absence of 3′ vector sequences, 834 bp product). Primer sequences are shown in Table 3, and amplification details described in methods. (**c**) PCR products of the six F0 pups with each of the four primer pairs run on 2% agarose gels/TAE. Controls include (−): B6 mouse DNA; (NT): non-template; (+): mix B6 plus donor vector. M: 1 kb Plus DNA Ladder (GeneRuler, ThermoFisher) (relevant gel bands encased in green boxes for easy visualization). Of the six pups, only female #5 fulfilled the four requisites of absence of 5′ and 3′ vectors sequences and presence of 5′ and 3′ loxP sites.

A 2 µl mix containing purified Cas9 (400 nM), selected 5′ and 3′ gRNAs (g79 and g92, 100 ng each), and the lssc form of the p*Mgp.Floxed* vector (20 ng) was injected into 69 embryos (out of 139 embryos obtained from 12 superovulated females). Ten embryos lysed during injections, and the remaining 59 were cultured overnight to yield 21, 2-cells embryos. The 21 embryos were implanted into one recipient CD-1 female mouse which produced 6 pups: #53, #54, #55, #56, #57, #58 (hereafter termed #1 to #6) (Fig. 4a). Genotype characterization of the pups was conducted by PCR according to the strategy depicted in Fig. 4b. Four primer pairs (P6f/P6r, P7f/P7r, P8f/P8r, P9f/P9r) were designed to identify the absence of 5′ vector sequences, the presence of the 5′- and 3′ loxP sites and the absence of 3′ vector sequences, respectively (Table 3). Presence of vector sequences would denote random integration of the donor DNA in the genome. Results of the DNAs from each of the six founders amplified with each of the primer sets are shown in gels (1), (2), (3) and (4) from Fig. 4c. Controls included DNA from the B6 mouse (negative), a non-template control, and a DNA mix of B6 plus donor vector (positive). Of the six pups, only one pup (#5) was simultaneously positive for 5′ loxP and 3′ loxP (505 bp and 324 bp bands in Fig. 4c gels (2) and (3) and negative for vector sequences (677 bp and 834 bp in Fig. 4c gels (1) and (4). The female with floxed allele, founder #5, was crossed 3X with a B6 male, and genotype of their F1 genome was reconfirmed with the same PCR primers and same strategy described above (presence of loxP sites and absence of vector sequences) (not shown).

**Table 3:** PCR primers for the screening of the F0.

Target site	Primer name	Sequence	Amplicon size (bp)
5′ vector junction	P6f P6r	5′ CGGGCCTCTTCGCTATTACG 3′ 5′ CGAGTGTTCTGGATGCAGTTTAATG 3′	677
5′ loxP site	P7f P7r	5′ GAGGAGTAATGTCAACCGAGGAGG 3′ 5′ TTAAGAGAAGTGTTCTCACCCCTTGGTA 3′	505
3′ loxP site	P8f P8r	5′ TGTGGCAACTACCTCGGGAGC 3′ 5′ AGGGCAAGGTGACTTCAGTG 3′	324
3′ vector junction	P9f P9r	5′ CTAGAAACAAGGCCTCGATCGGA 3′ 5′ TTTATGCTTCCGGCTCGT 3′	834

**Figure 5:**
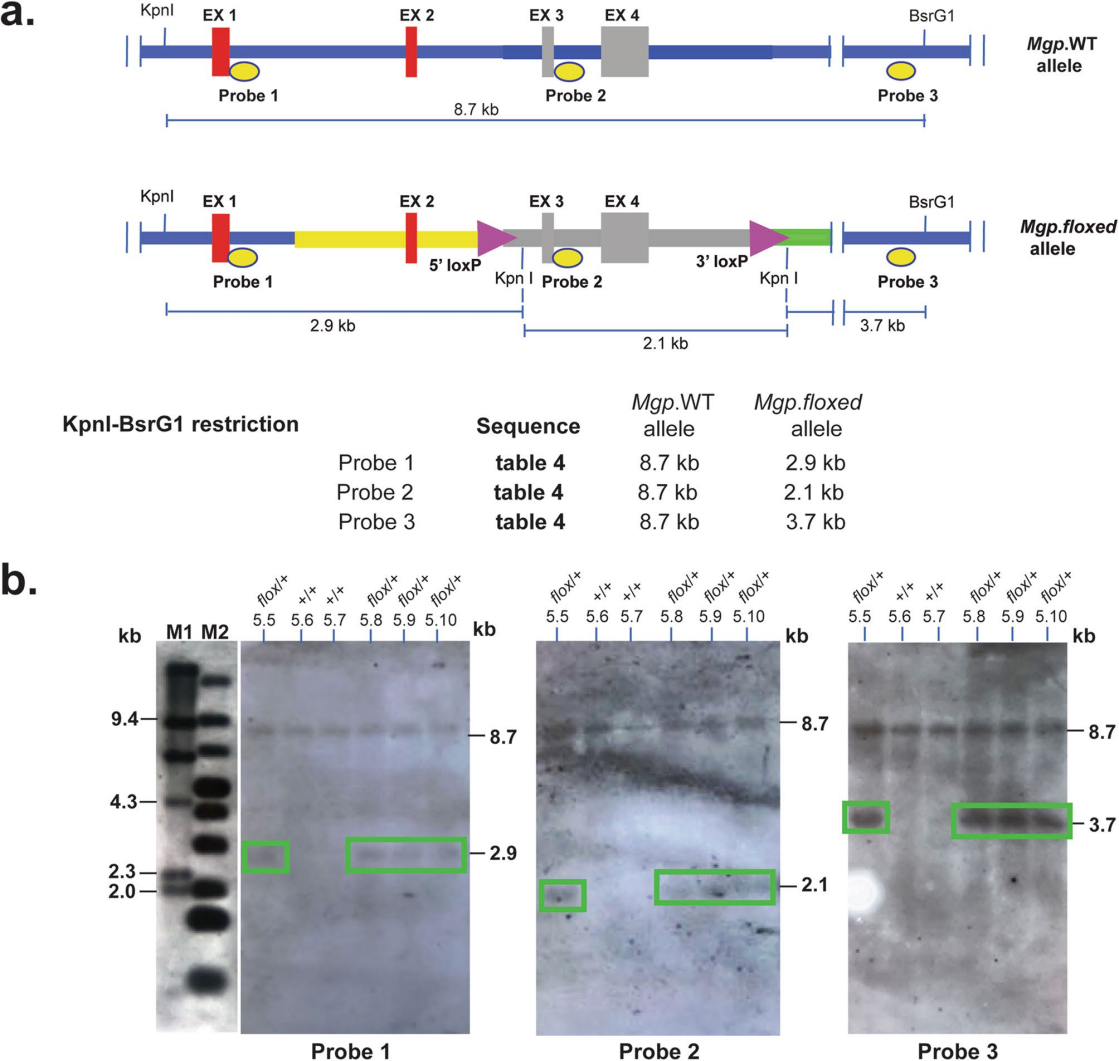
Confirmation of *Mgp.floxed* F1 genotype and loxP insertion. Female founder #5 was crossed with a B6 male and the genome of their six F1pups (#5.5 to #5–10) evaluated by southern slot hybridization. Tail DNA was digested with KpnI-BsrgI restriction enzymes, run in 0.7% agarose gels, blotted to a membrane and hybridized to three DIG-labeled oligonucleotide probes. Two of the probes located outside the target vector to assure correct genomic loxP insertion. The sequence of the probes and of the primers to generate them are shown in Table 4; experimental details are described in “Methods” section. (**a**) Diagrams of the *Mgp.*WT and *Mgp.floxed* alleles showing the location of the restriction enzymes, the three probes and the distance between the sites in kb. Expected fragment size in the both alleles upon the double enzyme digestion (note that KpnI sites were introduced during the generation of the donor vector, shown in Table 2); yellow and green original vector 5′ and 3′ homology arms. (**b**) Southern blot membrane containing the DNA from the six pups hybridized sequentially to the three probes after stripping in between hybridizations; relevant gel bands encased in green boxes for easy visualization. flox: floxed*.* M1: DIG-labelled DNA molecular markers II (La Roche); M2: 1 kb Plus DNA Ladder, which cross-reacts with the probes (GeneRuler, ThermoFisher). All genomic DNA fragments from both alleles showed the expected size. Mice # 5.6 and #5.7 were WT; mice #5.5, #5.8, #5.9 and #5.10 were heterozygous for the correct recombined allele.

Genotype of six F1 pups (#5.5, #5.6, #5.7, #5.8, #5.9 and #5.10) was additionally evaluated by southern blot hybridization (Fig. 5). Figure 5a shows the strategic design: three PCR-generated probes were made to uniquely identify the 5′, the internal, and the 3′ DNA fragments resulting from a KpnI-BsrgI digestion of the *Mgp.*WT and *Mgp.floxed* alleles (Fig. 5a). Sequences of Probe 1 (425 bp) obtained by amplification with P10f/P10r primers, Probe 2 (610 bp) amplified with primers P11f/P11/r, and Probe 3 (660 bp) amplified with P12f/P12r are shown on Table 4. Hybridizations of the digested WT allele rendered an 8.9 kb DNA band with the three probes. Because of the insertion of the KpnI sites with the loxP sequences (Table 2), hybridization of the digested *Mgp.floxed* allele with Probe 1 rendered the expected 2.9 kb band, while hybridization with Probe 2 and Probe 3 rendered 3.7 kb and 2.1 kb DNA fragments respectively. Mice #5.5, #5.8, #5.9 and #5.10 (2 males and 2 females) were heterozygotes for the *Mgp.floxed* allele (*Mgp*^*floxed*/+^), while mice #5.6 and #5.7 were WT (*Mgp*^+/+^) (Fig. 5b). This result confirms the sequential position of the 5′- and 3′-loxP sites on a single allele.

**Table 4:**
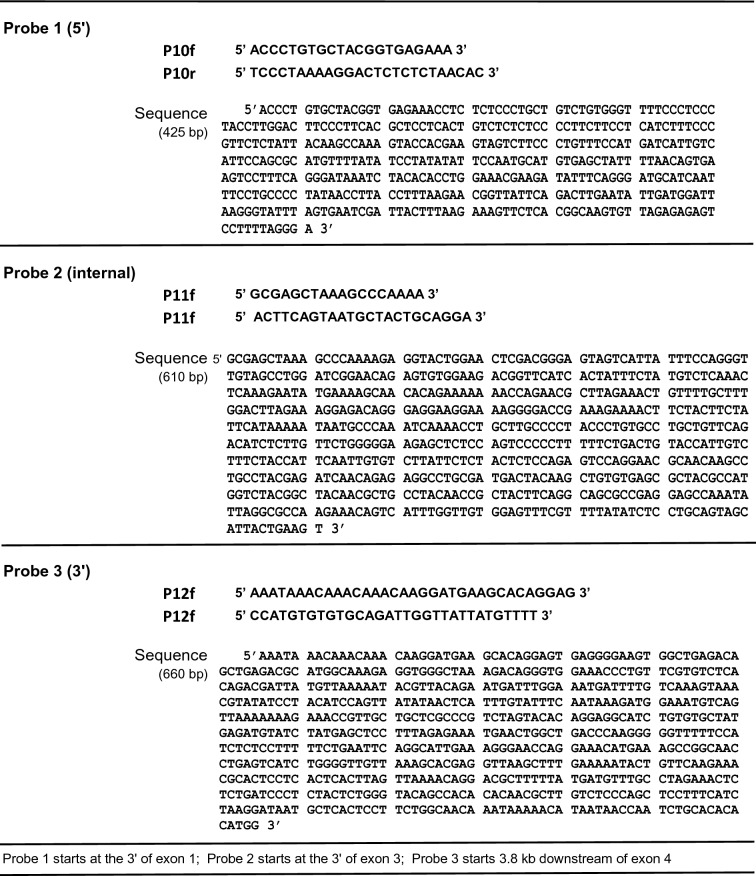
Generation and sequences of the probes used for southern blot characterization.

Probe 1 starts at the 3′ of exon 1; Probe 2 starts at the 3′ of exon 3; Probe 3 starts 3.8 kb downstream of exon 4.

Two intercrosses of the *Mgp*^*floxed*/+^ pups led to the generation of the first homozygous breeding pair *Mgp*^*floxed*/*floxed*^ mice. Litters from following generations of these mice were genotyped for about four generations with specific primers and subjected to the functional evaluation of the loxP sites in vitro and in vivo. A new optimized set of primers for the 3′ loxP site (P13f/P13r) which easily and clearly distinguished homozygous, heterozygous and WT genotypes was subsequently designed and used for genotyping (Fig. 1S).

### Vital and physiological characteristics of the *Mgp.floxed* mice

The *Mgp.floxed* (*Mgp*^*floxed/floxed*^) mice exhibit phenotypic characteristics which are indistinguishable from those of the parental B6. The *Mgp.floxed* mice are healthy, grow to normal weight size, reproduce with typical 5 to 8 pup littler size and have clear eyes. A longitudinal long-term IOP profile of this transgenic strain showed an initial 7.9 ± 0.2 mmHg at 2.5 month of age (n = 80), 8.2 ± 0.1 mmHg at 5 months (n = 20), and 9.7 ± 0.2 mmHg at 8 months (n = 20). These IOP values were not significantly different from those of the parental B6 strain at 3 months (8.1 ± 0.3 mmHg in parental (n = 8) versus 8.0 ± 0.2 mmHg in the *Mgp*.*floxed* (n = 20), *p* = 0.15.

### Validation of the *Mgp.floxed* mouse in vitro by Ad.GFP-2A-iCre infection of its primary iridocorneal angle cells

To validate the functional activity of the *Mgp*^*floxed/floxed *^loxP sites we directed expression of the Cre-recombinase protein to primary cells generated from the *Mgp.floxed* mouse*.* Because our primary interest is on eye tissues relevant to the development of glaucoma, we grew cells from the iridocorneal region of the mouse eye, which contain the trabecular meshwork (mouse iridocorneal angle cells, MIA cells). We generated these cells from both, the *Mgp.floxed* (MIA-F) and the B6 (MIA-B6) mice. Cre-recombinase was delivered by infecting subconfluent cells with adenovirus Ad.GFP-2A-iCre (hereafter Ad.GFP.Cre) at multiplicity of infection (moi) of 6–7 × 10^3^ viral genomes per cell (vg/cell). A second adenovirus, Adeno.GFP (hereafter Ad.GFP) was used for control at the same moi (Fig. 2S). For these experiments, four treatment groups (one experimental and three controls) were designed. Group 1 comprised MIA-F cells infected with Ad.GFP.Cre (experimental); Group 2 comprised MIA-F cells infected with non-Cre Ad.GFP (control); Group 3 comprised MIA-B6 cells infected with Ad.GFP.Cre (control); Group 4 contained MIA-F uninfected cells (control) (Fig. 2Sa). All experiments were performed in independently generated MIA cells lines, at passages 1 and 2 (see “Methods” section).

Two days post-infection, DNA, RNA, and protein from these cells were extracted and assayed for the presence of a floxed DNA fragment, levels of *Mgp* transcripts, and the absence of the Mgp protein. For the DNA evaluation, PCR primers P14f/P14r (sequence in methods) were designed to yield a 661 bp amplimer band of the floxed/recombined allele, and a 2804 bp of the unrecombined one (Fig. 2Sb). A second primer pair, P15f/P15r (392 bp, “Methods” section), was designed to be outside the *Mgp* loxP region of the gene and thus validate amplification levels of the treated and control templates. For evaluation of the *Mgp*-specific transcript levels, extracted RNAs from the treated and control dishes were reverse-transcribed and Taqman-PCR amplified with mouse *Mgp* Taqman probe annealing to exon 1–2 boundaries. The *Myoc* gene and 18S Taqman probes were used as controls. For protein evaluation, extracts were ran in PAGE gels and their transferred blots cross-reacted with a polyclonal Mgp antibody.

Results in Fig. 2Sc show that only DNA from the MIA-F/Ad.GFP.Cre infected cells (Group 1) showed the floxed 661 bp fragment (PCR gel, left). DNAs from control Groups 2, 3 and 4 showed instead the 2804 bp band in the PCR gel. No difference among the loaded DNAs of the four samples was found. For the RNA, and upon normalization with 18S, *Mgp* expression in Group 1 (experimental) was reduced 0.26 ± 0.02 of its expression in Group 4 (n = 2–3 cells lines per group). Group 2 and 3 were 1.0 ± 0.07 and 0.97 ± 0.09 of the expression in Group 4. In cells from Group 1, 2 and 3, the *Myoc* gene expression used for control was not reduced, with ratios of 0.97 ± 0.005, 0.87 ± 0.03, and 1.0 ± 0.0 from its expression in cells from UNT group 4, respectively. The abundance of the *Mgp* gene over the *Myoc* gene, normalized to 18S, was that of 484.4-fold. For the protein, only extracts from Group 1 cells lacked the 14 kDa Mgp protein (right) while those from control groups 2, 3 and 4 showed the presence of a robust Mgp protein in the western blot (right). The truncated protein, of estimated 1.5 kDa would not be detected in our gels. No difference among the loaded protein of the four samples was found. Uncropped western blots are shown in Fig. 4S.

Altogether these results indicate that the *Mgp* gene can be specifically recombined by Cre-recombinase in MIA cells originated from *Mgp*^*floxed*/*floxed*^ mice and demonstrate the correct insertion and functional activity of the loxP sites.

### Validation of the *Mgp.floxed* mouse in vivo by delivering Cre-recombinase to the trabecular meshwork by intracameral injection of Ad.GFP.Cre

Four groups of 2.5 to 3 months old mice (experimental and three controls) were used to determine whether the ablation of the *Mgp* gene occurred in the *Mgp.floxed* living mouse. To deliver Cre-recombinase to the targeted trabecular meshwork region, mice were injected intracamerally with 2 µl of adenoviruses Ad.GFP.Cre or Ad.GFP (1.5 × 10^9^ vg) using a 33G NanoFil syringe/microinjection system as indicated in “Methods” section. In parallel to the experimental design above in vitro, Group 1 comprised *Mgp.floxed* mice injected with Ad.GFP.Cre (experimental); Group 2 comprised *Mgp.floxed* mice injected Ad.GFP; Group 3 comprised B6 mice injected with Ad.GFP.Cre; Group 4 comprised *Mgp.floxed* mice uninjected.

**Figure 6:**
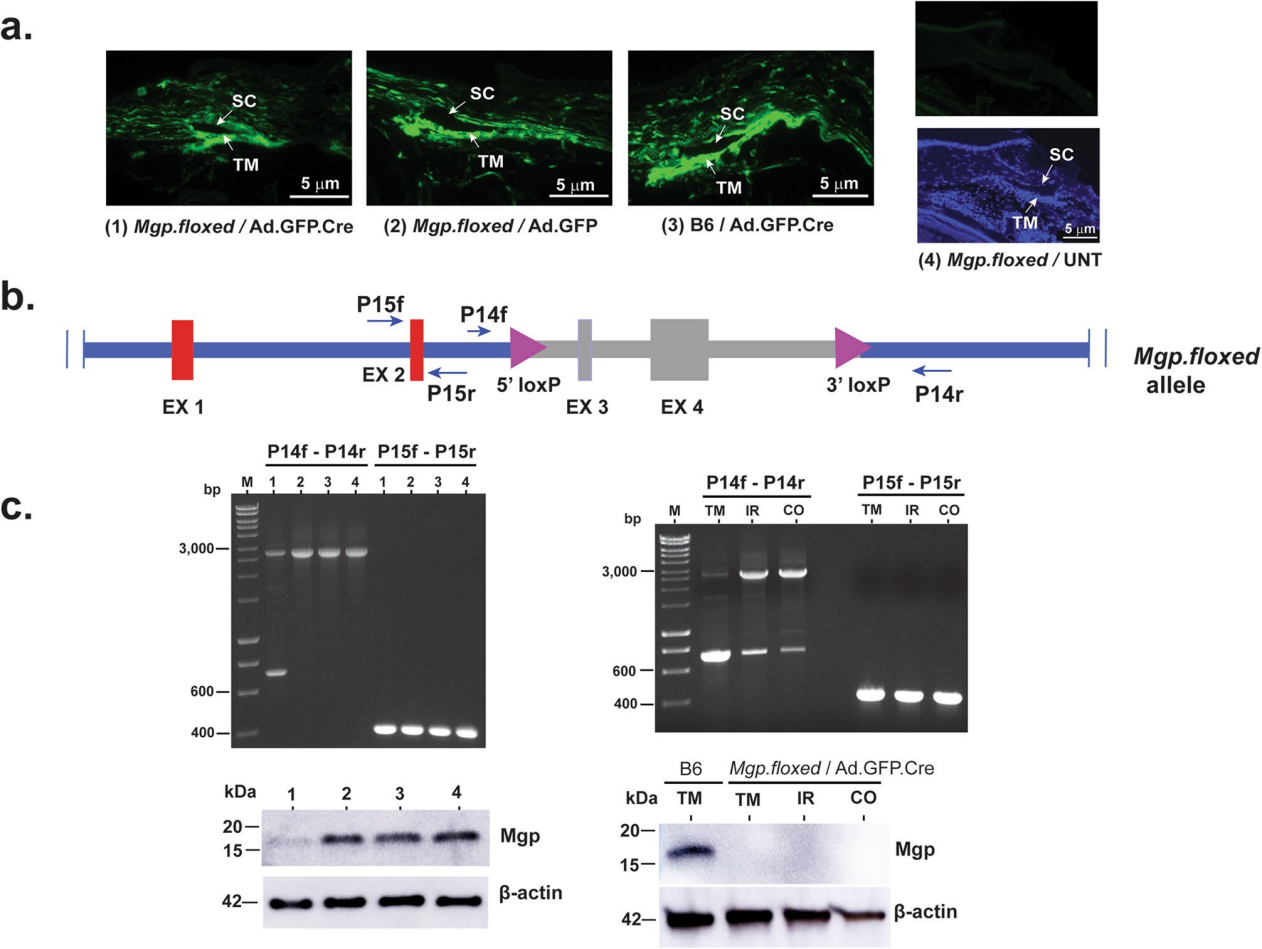
Characterization of loxP sites’ function on the *Mgp.floxed* mouse in vivo. Four groups of 2.5 to 3 months old mice were intracamerally injected with 2 µl (1.5 × 10^9^ vg) of Ad.GFP.Cre or Ad.GFP viruses or left uninjected. Group 1: *Mgp.floxed/*Ad.GFP.Cre-injected; group 2: *Mgp.floxed/*Ad.GFP-injected; group 3: B6 wild-type mice/Ad.GFP.Cre-injected; group 4: *Mgp.floxed/*UNT mice. (**a**) Representative images from 10 µm meridional anterior segments cryosections at 7 days post-injection. Groups 1, 2 and 3 showed GFP green fluorescence while uninjected group 4 (counterstained with DAPI) did not (n = 4 eyes per group). Transduction in all three infected groups was highly efficient. (**b**) Diagram of the *Mgp*.*floxed* allele. Target recombined region containing two exons is colored in gray. Arrows: primers to identify *Mgp* recombined DNA (P14f/P14r) and non-recombined control (P15f/P15r) regions. (**c**) Left (*top*): Representative1% agarose/TBE gel run with DNA extracted from iridocorneal strips dissected from single eyes from the four groups at 75 days post-injection (n = 3–5 eyes per group from 3 different BP). The *Mgp* gene is recombined (661 bp) only in the DNA extracted from group 1 while DNA from groups 2, 3 and 4 showed the 2804 bp band of the unrecombined gene. Internal control primers yielded an equal band of 392 bp in all four groups. (*Bottom*): Equivalent volumes of iridocorneal strips protein extracts from the four groups analyzed by western blot with anti-human MGP antibody (pooled 3 eyes/point, n = 2–3 per group). Membranes re-probed with anti-β-actin antibody as loading control. (**c**) Right (*top*): Similar PCR gel ran with DNA extracted from trabecular meshwork, iris and cornea tissues from *Mgp.floxed/*Ad.GFP.Cre-injected mice (pooled 3 eyes/point, n = 2). (*Bottom*): Similar western blot analysis ran with protein extracts from the same tissues (pooled 10 eyes/point), cross-reacted with anti-human MGP and anti-β-actin antibodies. B6 positive control (pooled 5 eyes/point). Only the trabecular meshwork from the B6 mice produces the Mgp protein. M: HyperLadder 1 kb (Bioline). TM: trabecular meshwork, SC: Schlemm’s canal, IR: iris, CO: cornea.

To first confirm positive gene delivery, a set of whole globes (n = 2 to 4 eyes per group), were embedded in OCT at 7 days post-injection to be able to observe GFP expression. Cryosections were evaluated for GFP fluorescence on an Olympus X71 microscope equipped with a DP80 monochrome camera. All injected eyes showed that the intracameral viral injections delivered the GFP protein mainly to the trabecular meshwork/Schlemm’s canal region (Fig. 6a). To then determine whether the *Mgp* gene was floxed/recombined in the angle tissue after Cre delivery, a second set of eyes was analyzed at 75 days post-viral injections using the eye globes from the IOP physiology experiments (see below). DNA was extracted from the iridocorneal angle strips of individual eyes and subjected to PCR amplification using the same P14 and P15 primer-pairs described above for the cells (Fig. 6b). Group 1 showed the *Mgp.floxed* allele recombined (661 bp fragment) at slightly lower full floxing efficiency of the 2804 bp unrecombined DNA. While in vitro we observed a complete floxing of the DNA, the slightly lower in vivo efficiency may be the result of the challenging technical delivery of the intraocular injection as well as of variations of the microsurgery of the trabecular meshwork strip, rather than to the ability of floxing the gene. No recombined DNA fragment was observed in control groups 2, 3 and 4. Likewise, a western blot with protein extracts showed the presence of Mgp protein only in control groups 2, 3 and 4 and not in the experimental Group 1 (Fig. 6c, left). Although it is known that the natural flow of aqueous humor delivers intracamerally injected viruses mainly to the trabecular meshwork, the extent of the differential delivery to other anterior segment tissues was not known. For this, we tested the presence of the *Mgp* floxed/recombined allele in all anterior segment tissues bathed by the aqueous humor. DNA and protein from trabecular meshwork, iris and cornea were extracted at 7 days post-injected whole globes, amplified and assayed by gel electrophoresis and western blot. Results shown in Fig. 6c right/*top* corroborated that the preferred targeted tissue after intracamerally injection of the Ad.GFP.Cre is the trabecular meshwork, which shows a close to complete recombination of the gene. It also shows that a small fraction of the virus enters the iris and the cornea and recombines their *Mgp.floxed* allele. Importantly, the iris and cornea tissues do not express the *Mgp* gene and thus, do not produce Mgp protein^38^ (Fig. 6c right/bottom). In all these experiments, controls with P15 pair amplifications and β-actin cross-reactive bands validated loading levels. In summary, this full characterization in vitro and in vivo of the *Mgp.floxed*/Ad.GFP.Cre mouse demonstrates the correct recombination of the *Mgp* in the trabecular meshwork and qualifies this mouse as a trabecular meshwork cKO (*Mgp.*TMcKO). Further, the availability of the *Mgp.floxed* mouse opens the door for the generation of permanent trabecular meshwork cKO through the use of genetic crossings, as well as to the generation of cKO for other tissues (local or otherwise) where the MGP protein plays a significant role.

### Ablation of Mgp in the trabecular meshwork in the *Mgp.*TMcKO mice results in elevated IOP

**Figure 7:**
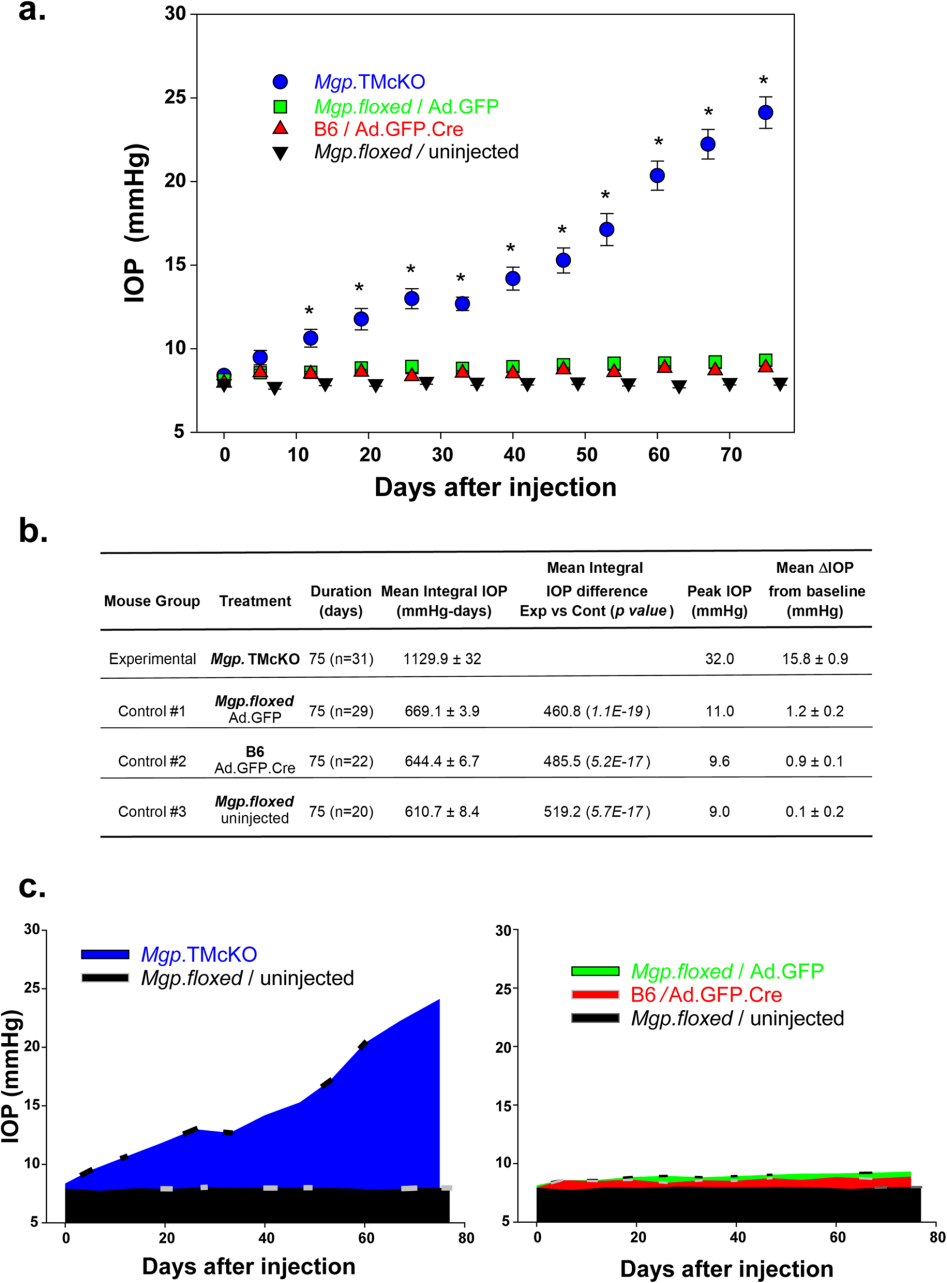
Absolute IOP profiles of the *Mgp*.TMcKO and control mice. *Mgp.floxed* mouse eyes were intracamerally injected with single doses of 1–5 × 10^9^ Ad.GFP.Cre viral genomes (n = 31) (blue), Ad.GFP (n = 29) (green), or left uninjected (n = 20) (black). One group of B6 mice was injected with same number of viral genomes of Ad.GFP.Cre (n = 22) (red). IOP was measured and averaged at pre-injection (day 0, baseline), and continued once weekly for 75 days. (**a**) IOP absolute values of treated and control eyes. Mean IOP of baselines of all eyes was 8.2 ± 0.11 mmHg. IOP of the *Mgp.floxed* group injected with Ad.GFP.Cre (*Mgp*.TMcKO) rose to 24.1 ± 0.19 mmHg at 75 days while that of the three control groups stayed at baseline values. The IOP of the *Mgp*.TMcKO eyes was highly statistically significant different from their own baseline (*p* = 1E−23) and from the three control groups at the same time period (*p* = 1E−18). (**b**) Summary table of the numeric values of measurements and calculations of IOP differences among the experimental *Mgp*.cKO and control groups. (**c**) Area under the curve (AUC) plots of the mean cumulative IOP experienced by each of the groups. *Left*: Integral IOP difference between the *Mgp.*TMcKO (blue) and *Mgp.floxed*/uninjected (black). *Right*: Integral IOP differences of the three control groups (*Mgp.floxed*/Ad.GFP (green), and *B6*/Ad.GFP.Cre (red) and *Mgp.floxed*/uninjected (black)*.* Ablation of the *Mgp* gene in the mouse trabecular meshwork resulted in elevated IOP.

The trabecular meshwork tissue of the eye is responsible for maintaining the physiological eye’s IOP by offering controlled resistance to the aqueous humor flow. In order to investigate whether the ablation of the Mgp protein affected the physiological IOP of mice, we produced a group of *Mgp*.TMcKO mice by injecting a single dose of Ad.GFP.Cre into the anterior chamber of *Mgp.floxed* mice. A total of 31 eyes of 2.5 to 3 months old mice of both sexes were intracamerally injected with 1–5 × 10^9^ Ad.GFP.Cre vg using the NanoFil syringe/microinjection system described in methods. Another three, age-matched control groups included *Mgp.floxed/*Ad.GFP-injected (n = 29 eyes), B6/Ad.GFP.Cre-injected (n = 22 eyes) and *Mgp.floxed/*uninjected (n = 20). After pre-injection baseline IOP measurements, IOPs were monitored weekly for 10 weeks using the TonoLab. The average IOP baseline values of all *Mgp.floxed* mice eyes used here (n = 80 eyes) was 8.2 ± 0.11 mmHg and it was not significantly different than that of the 8.0 ± 0.10 mmHg baseline of the B6 (n = 22) (*p* = 0.31). The mean absolute IOP of the *Mgp.*TMcKO started to increase at 5 days post-injection and became significant at 12 days. Pressures continued to increase steadily for 75 days, when the experiment was terminated (Fig. 7a). At the same time-period, pressures of the eyes from the three control groups remained at baseline levels. Thus, at 40 days, while the mean IOP of *Mgp.*TMcKO was 14.2 ± 0.7 mmHg, the mean IOPs of the controls were 8.9 ± 0.1 mmHg (*Mgp.floxed*/Ad.GFP), 8.5 ± 0.1 mmHg (B6/Ad.GFP.Cre) and 8.0 ± 0.1 mmHg (*Mgp.floxed*/uninjected) respectively (*p* < 4E−9 on all *Mgp.*TMcKO vs controls comparisons). At the end of the experiment, the *Mgp.*TMcKO’s IOP rose to 24.1 ± 0.9 mmHg while those of the three controls remained on 9.3 ± 0.1, 8.9 ± 0.1 and 8.0 ± 0.2 mmHg at baseline values (*p* < 1E−18 on all *Mgp.*TMcKO vs controls comparisons) (Fig. 7a).

Additional IOP parameters from the four groups are shown in Fig. 7b,c. The mean integral IOP (area under the curve, AUC) (defined as the mean of the cumulative IOP that each eye received for the duration of the experiment) was 1129.9 ± 32 mmHg-d for the experimental *Mgp.*TMcKO group (Fig. 7b,c). For the three control groups, at the same time-period, the mean integral IOPs were 669.1 ± 3.9, 644.4 ± 6.7 and 610.7 ± 8.4 mmHg-d respectively (Fig. 7b,c). The mean integral IOP difference between experimental and control groups was highly significant for all comparisons (Fig. 7b,c). Considering the whole study period, the Peak IOP in the *Mgp.*TMcKO mice group at 75 days was 32 mmHg (full range of all eyes 14.0 to 32 mmHg). For the three control groups, at the same time period, the Peaks’ IOP were 11.0 mmHg (range 8.3 to 11 mmHg), 9.6 mmHg (range 8.3 to 9.6 mmHg) and 9.3 mmHg (range 7.0 to 9.3 mmHg) respectively. At the last 75 days point, the mean ΔIOP from baseline was 15.8 ± 0.9 mmHg for the *Mgp.*TMcKO while those of the controls were 1.2 ± 0.2 mmHg for control *Mgp.floxed*/Ad.GFP, 0.9 ± 0.1 mmHg for control B6/Ad.GFP.Cre and 0.1 ± 0.2 mmHg for control 3 *Mgp.floxed*/uninjected. The eye with the highest IOP difference (ΔIOP of each eye between its IOP at 75 days and own baseline) for the *Mgp.*TMcKO experimental group was 23.4 mmHg. For the three control groups, at the same time period, the eyes with the highest IOP differences were 3.3, 2.5 and 1.3 mmHg respectively.

Altogether these results indicate that the *Mgp* gene is both, responsible and sufficient for maintaining the physiological pressure in the mouse eye.

## Discussion

To the best of our knowledge, and despite the relevance of the *MPG* gene in an increased number of human diseases, no *Mgp.floxed* animal model has to date been generated. In this study, we show that a stepwise strategy led to the successful construction of the *Mgp.floxed* mouse. The generation of this mouse is particularly important because the *Mgp.*KO is lethal and the only way to study the function of the gene in specific post-natal tissue is by generating tissue-specific cKOs and ablating the gene locally. We attribute the success of the first F0 with floxed allele *Mgp*^*floxed/*+^ mouse to a combination of events that included selecting the correct loxP insertion sites in the *Mgp* gene, low off-target gRNAs, and the construction of a singular donor plasmid. This particular donor plasmid contained a 3802 bp *Mgp* donor DNA fragment which included both 5′ and 3′ loxP sites *in cis,* inserted restriction sites for subsequent validation of the mouse, and long homology arms to facilitate accurate integration. The inserted restriction sites allowed confirmation of the accurate recombination in the F1 generation. Based on our previous failed attempts (Table 1S), in this study we injected the embryos with two gRNAs, the purified Cas9 protein, and the entire plasmid donor DNA (6.7 kb) in a single-stranded circular form. This long-single-stranded-circular DNA was generated by using a vector-specific gRNA, a mutated Cas9 and the nuclease III 3′ end activity. Our rationale for using the lsscDNA was that the published methods for generating long linear SSDNA involved error-prone synthesis methods and raised the concern that unwanted mutations might be co-introduced with the loxP sites. However, we really don’t have the data to emphasize this method as being novel and better than other donor methods. The use of a long-single-stranded linear DNA (lssDNA) had previously been reported to increase efficiency of obtaining floxed alleles^10,52,53^ with such efficiency depending on its length^52,54^. In here, a much larger donor plasmid DNA fragment (3.8 kb) contained in a ss circular DNA donor vector, resulted in the correct floxed allele. At this time though, we are unable to say whether a contaminant double-stranded molecule in the lssc preparation, could have contributed to the generation of the *Mgp.floxed* allele.

Because of the high abundance of the *Mgp* gene in the trabecular meshwork/outflow pathway tissue^29–31^, of its expression induction by glaucomatous-causing agents^34–36^ as well as its involvement in IOP homeostatic response^35^, our laboratory has had a long-time interest in identifying the functional role of this gene in this tissue^36,45,55^. It is well established that dysfunction of the trabecular meshwork in its ability to regulate IOP leads to the development of glaucoma, a blinding disease with enormous global health repercussions. Until now, eye studies on this gene have mainly been conducted using primary human cells and organ cultures but not in living animals. Our first transgenic mouse, *Mgp-*Cre.KI^38^ confirmed in vivo the localized high expression of the gene to the eye outflow pathway region. To focus now on function, in this study we generated a second mouse, *Mgp.floxed*, which would allow us to subsequently generate an *Mgp.*TMcKO. For this, and given our ability to deliver genes to the trabecular meshwork by intracameral injections in different animal species^56–59^, we chose an adenovirus vector to target the Cre-recombinase enzyme to the trabecular meshwork. Although adenovirus delivery expresses the transgene for a short, limited time we show here that 7 days post-injection appeared to be sufficient for the delivered enzyme to recombine most of the trabecular mouse genomic DNA in vivo (Fig. 6). Concurrently, we also saw that a very low proportion of the virus entered surrounding tissues facing the anterior chamber other than the trabecular meshwork. Iris and cornea DNA were minimally recombined. This finding (Fig. 6c-right), together with our previously published result that *Mgp* is not expressed in the iris nor cornea^38^, validated the intracameral injection route of administration of the Ad.GFP.Cre as a reliable system for the generation of the *Mgp.*TMcKO.

The *Mgp.*TMcKO developed elevated IOP. We observed a steady IOP increase in the mouse cohort injected with the Ad.GFP.Cre while no increase was observed on any of the uninjected cohorts of the *Mgp.floxed* mice, of the *Mgp.floxed* mice injected with Ad.GFP or of wild type mice B6 injected with Ad.GFP.Cre. The increase was gradual. It started at 5 days post injection, became significant at 12 days and continue to increase to 75 days, when the experiment was terminated. Because the biochemical characterization of the trabecular meshwork tissue of the Cre-treated mice showed the absence of the Mgp protein, these results strongly indicate that the presence of *Mgp* appears to be essential for the maintenance of physiological pressure in the mouse eye.

The potential mechanisms as to how the specific ablation of this gene in the outflow pathway leads to an elevated IOP in the mice are currently being investigated. The fact that the observed increase of pressure occurs gradually is an indication that the *Mgp* gene is not causing a sudden obstruction of the outflow pathway but rather causes a metabolic cellular change whose accumulation would lead to changes in the ECM that increase IOP. Given the classical known function of *Mgp* as an inhibitor of calcification, one would first suspect that the outflow tissue without this gene would undergo an extracellular calcification process which in turn could be translated in an increase of stiffness. It has been established that the trabecular meshwork from glaucomatous patients is stiffer than that of the controls^60^, and that glaucomatous effectors such as TGFβ2 induce stiffness^55,61^. The calcification process in the vascular system is classified in macrocalcification and microcalcification depending on the pattern and size of the calcified lesions^62^. In atherosclerosis, macrocalcification is manifested by the formation of large calcified lesions (plaques) while microcalcification is associated with spotty, granular calcification and the progress of the disease^63^. While macrocalcification is easily observed by classic VonKossa and Alizarin Red stainings, microcalcification would require specific staining methods. It would seem likely that the gradual increase of IOP observed here in the *Mgp.*TMcKO would be more in tune with the presence of a microcalcification rather than a large calcification lesion.

Mgp is secreted in matrix calcifying vesicles. Another interesting possibility would be that of the involvement of *Mgp* in maintaining the extracellular calcium homeostasis, a recently described action of the gene shown to occur during sperm maturation^33^. In the trabecular meshwork, Mgp is carboxylated, therefore active. In the sperm, it was shown that uncarboxylated, inactive MGP which loses its ability to bind calcium, contributes to high Ca concentrations in the epididymal lumen^33^. It would be interesting to determine whether the absence of Mgp in the outflow pathway cells could also disturb the Ca levels on the ECM which could in turn affect extracellular calcium entry and modulate trabecular meshwork function^64^. Another potential mechanism responsible for the elevated IOP in the *Mgp.*TMcKO could involve BMP2. It is well established that MGP binds to, and sequesters BMP2^36,65,66^. To this end, we had earlier shown that overexpression of BMP2 in rat trabecular meshwork by intracameral injection of Ad5BMP2 induced elevated IOP^67^. An increase in BMP2 triggered by the absence of *Mgp* could alter the physiology of the trabecular meshwork through several pathways. As reasoned in Buie et al.^67^, besides provoking an increase in calcification^68,69^, BMP2 could influence IOP by its consequent increases in *Col1A1*^67^, a trabecular meshwork ECM component, originally reported to be enhanced under glaucomatous conditions^70^. More recently *Col1A1* has been found to be genetically linked to glaucoma cohorts^71^. In the same Buie’s paper^67^, it was also discussed the well-known crosstalk between BMP2 and the Wnt/β-catenin pathways and the activation of each of the pathways by the other^72,73^. There is an apparent paradox though, that while BMP2 activates the Wnt/β-catenin pathway^73^ and causes elevated IOP^67^, an antagonist of the Wnt/β-catenin pathway, SFPR1, causes also elevated IOP^74^. However, some studies have shown that BMP2 can also inactivate Wnt/β-catenin^75^ and that its activation or inhibition of the pathway is dependent on the status of the expression of p53 and SMAD4 tumor genes, like in colorectal cancer cells^76^. It would seem then plausible that in the trabecular meshwork, BMP2 could be inhibiting the Wnt/β-catenin pathway.

The close relationship of MGP with elastin brings another interesting avenue on to the potential role of *Mgp* in maintaining tissue integrity and physiological IOP. On the eye’s outflow tissue, elastin is an essential component of the trabecular meshwork ECM^77^. Together with collagens and proteoglycans, elastin forms part of the beams of the corneoscleral region upon which lie the trabecular meshwork cells. A network of elastin-like fibers is also present in the juxtacanicular region and the thickness of these fibers has been associated with glaucomatous specimens^77^. Findings in the vascular and chondrocytes/osteoblast fields have demonstrated that elastin functions as the scaffold of mineralization. Further, MGP colocalizes with elastin in the arterial elastic lamina which is the first site of ectopic mineralization in the *Mgp.*KO^78^. It is also known that there is a correlation between calcification and degradation of elastic fibers^79^ and that elastic fragmentation precedes vascular calcification^80,81^. A recent finding further showed that an *Mgp.*KO mouse with elastin haploinsufficiency (*Mgp*^*−*/*−*^; *Eln*^+*/−*^) exhibited reduced calcification^82^. It is intriguing that elastin degradation is also the hallmark of Marfan’s syndrome, a connective tissue disease caused by a mutation in fibrillin1, a protein essential for the formation of the elastic fibers^83^. About 2% of Marfan’s patients exhibit glaucoma^84,85^. Moreover, elastin degradation and fragmentation led to the release of elastin peptides, which colocalized with areas of microcalcification in the aorta of Marfan’s patients^86^. Altogether, these data from other systems bring out the possibility that the absence of Mgp in the trabecular meshwork could induce elevated IOP by just inducing an alteration on its ECM’s elastic network^82^. Elastin fragmentation followed by initiation of microcalcification could further contribute to ECM changes that could lead to increased resistance to aqueous humor outflow facility. These changes in the elastic network would affect the structure and integrity of the trabecular meshwork tissue which is so critical for the maintenance of physiological pressure.

In summary, in this study we have used a combination of strategies to generate an *Mgp.floxed* mouse (*Mgp*^*floxed/floxed*^). The use of a plasmid with a 3.8 kb donor DNA containing long homology arms, the 5′ and 3′ loxP sequences *in cis,* and its conversion to a single-stranded *circular* form before microinjection, together led to the success obtaining a floxed founder animal. The functional loxP recombination and ablation of the gene, fully characterized by viral gene delivery of the Cre-recombinase enzyme, opens the door to the use of this mouse to study *Mgp* roles in different tissues and diseases. Using this approach for the study of glaucoma, we report here the generation of the first *Mgp.*cKO in the trabecular meshwork, which is the tissue responsible for the regulation or intraocular pressure in the anterior segment of the eye. We find that the absence of Mgp protein in this tissue resulted in elevated IOP in the living animal. These findings show that the presence of Mgp is essential to maintain the physiological IOP and protects the eye for developing elevated pressure. They have uncovered a new function for the *Mgp* gene*.* The mechanisms by which *Mgp* is responsible for maintaining eye pressure are yet to be investigated. Whatever those might be, *Mgp* appears as a good therapeutic target for the treatment of glaucoma.

## Methods

### Mice

All animal work was performed as approved by the Institutional Animal Care and Use Committee at the University of North Carolina at Chapel Hill (UNC) and conducted in accordance with the ARVO Statement on the Use of Animals in Ophthalmic and Vision research. All animals were housed in temperature-controlled rooms under standard 12-h cycle lighting with food and water provided ad libitum. The mouse strain used to obtain the embryos was C57BL/6J (B6) (Jackson Laboratory, Bar Harbor, ME, USA 000664). The mouse strain used to implant the blastocysts was CD-1 (Charles River Laboratory, Wilmington, MA, USA 022). The mouse strain used for experimental controls was also B6.

### Vector constructions for the preparation of Cas9, gRNA and lssc donor DNA

For the production of the Cas9 protein, a human codon optimized Cas9 cDNA^13^ (Addgene 41815), engineered with a His6 tag at the C-terminus, was cloned into the pET-28a(+) vector (Novagen/Sigma-Aldrich, St. Louis, MO, USA 69864) at NcoI/Eco53ki sites and transformed into BL21(DE3) competent cells (New England Biolab, NEB, Ipswich, MA, USA). The expression of Cas9 was induced with IPTG for 4 h at 25 °C. The Cas9 protein was purified using Ni–NTA agarose (Qiagen, Germantown, MD, USA) and dialyzed against 20 mM HEPES (pH 7.5)/150 mM KCL/1 mM DTT/10% glycerol^87^.

For the generation of plasmids pT7.gRNA.g79/92, paired oligos (Table 1) were annealed in 200 µl on final concentrations of 1 µM each in a thermocycler (95 °C 3 min, 95 °C (− 1 °C per cycle) for 69 cycles, hold at 4 °C) and saved annealed at − 20 °C until use. Parent T7 vector was digested with BsaI and 50 ng of the restricted vector were incubated with 18 µl of the annealed solution and 1 µl of T4 ligase (NEB) for 10–15 min at room temperature in a final of 20 µl. Aliquots of the ligation reaction were used to transform Stellar competent cells (Takara Bio/Clontech, Mountain View, CA USA) by standard procedures and plated on kanamycin plates. gRNAs were transcribed from linearized plasmids using HiScribe T7 High Yield RN Synthesis kit (NEB, E20540S) and RNA purified using RNeasy columns (Qiagen).

For the generation of the plasmid donor vector (p*Mgp.Floxed*) In-Fusion cloning was conducted with three DNA elements (5′ homology arm, donor fragment and 3′ homology arm) using an In-Fusion HD cloning kit (Takara 639649). The three DNA elements were amplified from genomic B6 DNA obtained from tail tips by the Hot Shot method^88^. The elements expand an *Mgp* region from 226 bp upstream of exon 2 to 1655 bp downstream of exon 4 (total 3802 bp including loxP sites and added restriction enzyme sites). They were amplified with primers containing 15 bp end sequences overlapping the EcoRV/BamH1 linearized pL453 vector and each other, plus the desired insertions of loxP and restriction enzymes sites sequences (Table 2). Amplifications were conducted using Q5 HD polymerase, 5 × reaction buffer and 5 × GC buffer (NEB M0491L), 0.4 µM corresponding primers and 20 ng of genomic DNA, at 98 °C 3 min, 95 °C 30 s, annealing temperature 30 s. (5′ element 58 °C, middle donor fragment 64 °C, 3′ element 70 °C), 72 °C 1 min, 35 cycles. DNA fragments were purified by gel electrophoresis. The 5′ element (100 ng), the middle donor fragment (100 ng), the 3′ element (100 ng), and the linearized and purified pL453 backbone (120 ng) were combined in one 10 µl cloning reaction with the In-Fusion HD Cloning Plus enzyme mix (Takara 638920) at 50 °C for 15 min. A control reaction lacking the DNA from the three elements was conducted in parallel. Transformation was conducted on Stellar cells (Takara) and colonies grown on ampicillin plates. Isolated colonies were amplified, and their plasmid DNA confirmed by sequencing. One sequenced confirmed plasmid, p*Mgp.Floxed* was grown and purified using a maxiprep kit (Qiagen 12963).

For the preparation of the injection-grade lssc DNA, 50 µg of p*Mgp.Floxed* DNA were incubated at 37 °C for 60 min with a newly designed gRNA containing matching vector sequences, and a D10A mutated single-stranded cutting Cas protein (hCas9_D10A was a gift from George Church (https://n2t.net/addgene:41816; RRID:Addgene_41816) to produce nicked-p*Mgp.Floxed*. The gRNA and the mutated Cas protein were cloned and produced from the pT7RNA and pET-28a(+) plasmids as indicated above. Forty 40 µg of nicked p*Mgp.Floxed* DNA were incubated with 20 µl (100 units/µl) Nuclease III (NEB M0206) for 30 min at 37 °C, purified by a quick spin column and dialyzed.

### Embryo injection and mouse production

Mouse embryos were collected from the oviduct of naturally mated B6 females that were superovulated by injection with PMS (ProSpec, East Brunswick, NJ, USA) and HCG (Cosmo Bio USA, Carlsbad, CA, USA). A 1 µl mix containing purified Cas9 (400 nM), 5′ and 3′ gRNAs (gRNA g79 and gRNA 92, 50 ng each), and the lssc form of the p*Mgp.Floxed* vector (30 ng) was prepared and from there, a few pl were injected into the embryo pronucleus with a micromanipulator. The injected embryos were cultured in KSOM media (CytoSpring, Mountain View, CAL USA) overnight and embryos that developed to the two-cell stage were transferred into the oviducts of pseudopregnant females.

### Genotyping of neonatal mice (F0 and F1)

Genomic DNA was extracted from the tail tip using the Hot Shot method indicated above^88^. For PCR, we used four primer pairs designed to target the inserted region externally and internally (Table 3). PCR was performed in a total volume of 25 µl at 95 °C 2 min (95 °C 30 s, 72 °C 30 s (− 1 °C per cycle), 72 °C 1 min) for 14 cycles, then (95 °C 30 s, 58 °C 30 s, 72 °C 1 min for 24 cycles), and ending at 72 °C for 2 min using ThermoPol mix (NEB). Two of the four primers were used to identify the correct insertion of the loxP sites, while the other two were used to identify the presence of 5′ and/or 3′ vector sequences in the genomic DNA. Each of the four PCR products from the DNA of the six F0 neonatal mice was run in a 2% agarose/TAE gel. Negative controls included DNA from a parental non floxed mouse and a non template reaction. Positive control included the vector DNA.

For the southern blot genotyping of the F1, tail tip genomic DNA was isolated using standard proteinase K extraction method (200 µl of 30 mM Tris/10 mM EDTA/1% SDS/12 µl of 10 µg/ml proteinase K followed by ethanol precipitation). Each pup DNA (10 µg) was digested with KpnI/BsrG1 overnight and run on a 0.7% agarose gel also overnight. After denaturation, the gel was blotted to a nylon membrane (Roche Life Science, Branford, CT, USA) by capillary action for 16 h. The blot was hybridized subsequently to three, DIG-labeled oligonucleotide probes in Hybing solution (DIG Easy Hyb, Roche) at 45 °C in rolling bottles for 16 h. The three oligonucleotide probes (Table 4), each specific for a region of the DNA, were obtained by PCR amplification in a total volume of 25 µl. Conditions were 95 °C 2 min, (94 °C 30 s, 72 °C 30 s (− 1 °C per cycle), 72 °C 1 min) for 14 cycles, then (95 °C 30 s, 58 °C 30 s, 72 °C 1 min) for 24 cycles, and ending at 72 °C for 2 min using the PCR DIG probe synthesis kit (Roche 11636090910). After hybridization, blots were sequentially washed with 2 × SSC at room temperature and 0.5 × SSC at 65 °C, exposed to X-ray film (Genesee Scientific, El Cajon, CAL USA). For stripping in between hybridizations, blots were incubated 2 × in stripping buffer (0.2 M NaOH, 0.1% SDS) at 37 °C for 15 min each. DNA Molecular Marker II Dig labelled (Roche) and 1 kb Plus DNA Ladder (GeneRuler, ThermoFisher, Waltham, MA, USA) were used to determine the sizes of the hybridized fragments*.*

Once all identification was complete and *in cis* arrangement was established, a simplified genotyping priming pair was designed to identify homozygous *Mgp*^*floxed/floxed*^, heterozygous *Mgp*^*floxed/*+^ and wild-type *Mgp*^+*/*+^ alleles. Primers P13f: 5′ AAGGTAGGGAGCCCATGACAGGTC 3′ and P13r: 5′ TCGGGAGAGGAACAAGCTTCGCCTGT 3′ amplified a 358 bp (WT) and 300 bp (floxed) fragments which were clearly separated in a 2% agarose/TBE gel (Fig. 1S).

### Primary culture of mouse iridocorneal angle cells (MIA cells)

Mice 2–4 months old were euthanized by an overdose intraperitoneal (IP) injectable anesthesia (400 mg/kg ketamine/20 mg/kg xylazine/4 mg/kg acepromazine) (Covetrus, Dublin, OH, USA), followed by cervical dislocation immediately prior to tissue collection. Whole globes were enucleated, cleaned and washed with PBS. Under a dissecting microscope, globes were then bisected a few mm posterior to the limbus using an Optical microsurgery blade (Wilson Ophthalmic, Mustang, OK) and iridectomy scissors. After removing the lens, separated anterior segments were cut into four quadrants and iris and ciliary body carefully removed with forceps. Strips of the angle region containing the trabecular meshwork were obtained by making anterior and posterior incisions of the iridocorneal region and placed them on 2% porcine gelatin-coated (Sigma) 35 mm dishes. The tissue was coverslipped with a drop of MEM Richter’s Modification medium (IMEM, HyClone/ThermoFisher) supplemented with 20% fetal bovine serum (FBS, Gibco/ThermoFisher), 50 µg/ml gentamicin (Gibco/ThermoFisher) and cells allowed to grow for 3–4 weeks changing the media every other day. Upon confluency, cells from the right and left eyes of the same mouse were trypsinized, pooled, passed to 60 mm dishes to confluency, and either harvested and stored in liquid nitrogen, or used directly for the experiment. In total, the MIA cells used in this study originated from six *Mgp*.*floxed* mice (6 cell lines, MIA-F1, 2, 4, 5, 6 & 7 from 6 different breeding pairs) and from two B6 mice (MIA-B61 and MIA-B62, from 2 different breeding pairs). All cells were used at passages 1–2.

### Adenoviral vectors and infection of MIA cells

Recombinant adenoviruses Ad-GFP-2A-iCre and control Adeno.GFP were obtained commercially (Vectors Biolabs, Malvern, PA, USA, and Qbiogen, Carlsbad, Canada, respectively), grown and purified in our laboratory^58^. The Ad-GFP-2A-iCre (termed Ad.GFP.Cre in the manuscript) expresses both a codon improved Cre-recombinase (iCre) an eGFP marker. Cre and GFP are driven by the same CMV promoter and separated by 2A peptides. The Adeno.GFP (termed Ad.GFP in the manuscript) is an adenovirus 5 carrying a variant of the jellyfish Aequorea vitoria GFP driven by the CMV promoter^58^. Physical particles were tittered as viral genomes (vg)/ml by extracting an aliquot of purified viral DNA (DNeasy Blood and Tissue kit, Qiagen 69054), measuring its optical density (Nanodrop One, ThermoFisher) and converting each ng of DNA to the number of DNA molecules based on its MW. Viral infectivity (plaque-forming units per ml, pfu/ml) was measured with a QuickTiter Adenovirus Titer Immunoassay kit (Cell Biolabs, San Diego, CA, USA VPK-109) following manufacture recommendations. Viral lots used in this study had concentrations of 5–6 × 10^11^ vg/ml (0.2–2 × 10^11^ pfu/ml) (Ad.GFP.Cre) and 5 × 10^11^ vg/ml (3.9 × 10^10^ pfu/ml) (Ad.GFP) respectively.

MIA primary cells at passage 1 to 2 seeded on six-well dishes were grown to 70% to 80% confluency, washed twice with PBS, and exposed to the recombinant adenoviruses in 1 ml serum-free medium. After exposure to the virus for 2 h, complete media was added, and incubation continued for 2 days. Fluorescence images were captured on living cells with an inverted IX71 Olympus fluorescence microscope equipped with a DP80 monochrome camera and cellSense software (Olympus, Center Valley, PA, USA).

### Intracameral microinjection of recombinant viral vectors

The microinjection system for nanoliter-accurate delivery to the intracameral space of the mouse consists of a 10 µl specially designed glass syringe (NanoFil syringe, World Precision Instruments, WPI, Sarasota, FL, USA) mounted on an UltraMicroPump (UMP3, WPI) which is connected to a small controller box (Micro2T SMARTouch, WPI) to program delivery conditions. The NanoFil syringe is attached to a quartz flexible tubing (SilFlex) (35 cm long, 100 µm ID, 460 µm OD), reinforced at both ends with teflon jackets. One end of the Silflex tubing was connected to the NanoFil syringe, while the other end was connected to a 1 cm PE-10 intramedic tubing piece (0.38 mm ID, Clay Adams, Parsippany, NJ, USA) which in turn was tightly connected to the 460 µm OD shank of the 33G NanoFil needle (total 40 mm long). This modification allowed to hold the NanoFil needle with a needle holder (Barraquer, Storz Ophthalmic Instruments, Rochester, NY, USA) and to more accurately drive it to the injection site. The Micro2T SMARTouch controller box was programmed to deliver 2 µl in 30 s (67 nl/s). The overall system is depicted in Fig. 3S.

Each mouse was anesthetized by an IP injectable anesthesia (50 mg/kg ketamine/5 mg/kg xylazine/1 mg/kg acepromazine) (Covetrus). Whiskers were trimmed and pupils dilated with a drop of 1% tropicamide ophthalmic solution (Covetrus). While resting slightly on its side with its tail to the right, the mouse was placed under a surgical stereo microscope (Leica M80) equipped with a DFC450 digital camera (Leica Microsystems, Buffalo Grove IL, USA). The NanoFil syringe was filled with the different viral treatments and the mouse eye was secured nasal to temporal with fine Straight Bishop-Harmon Tissue forceps (Storz E1500). These forceps, once closed at its front, leave a space between the shafts that helps protect the optic nerve from crushing when holding the eye during the injection. The NanoFil needle was inserted through the cornea a few mm from the retracted iris by holding the needle with the needle holder. To facilitate entrance, the cornea was superficially pricked with a 27G needle prior to the NanoFil needle insertion. When the NanoFil needle was inside the anterior chamber, the UMP3 micropump was turned on by the Micro2T box controller, and fluid entry monitored by direct visualization through the operating microscope. After delivery, the NanoFil needle was left in place for 30 s. and withdrawn gradually to minimize leaking. Topical antibiotic ointment (neomycin 3.5 mg/g, polymyxin B 10,000 U/g, and bacitracin 400 U/g) (Covetrus) was applied to the eyes, and animals returned to their cages, resting on heating pads for recovery.

### RNA extraction, reverse transcription and TaqMan-PCR assays

Primary MIA cells were scraped from tissue culture dishes with guanidine thiocyanate buffer (RLT, Qiagen). Total RNA was extracted by loading the solution onto a QIA Shredder column and continued by the use of the RNeasy Plus Mini kit with gDNA removal column according to manufacturer’s recommendations (Qiagen 74134). Purified RNA was eluted in 30 µl RNase-free water and the concentration measured in a NanoDrop One (ThermoFisher). Total RNA recoveries averaged 3–4 µg per 35 mm culture dishes respectively.

Reverse transcription (RT) reactions were conducted with 1 µg primary MIA cells RNA in a 20 µl total volume of proprietary RT buffer at 25 °C 10 min, 37 °C, 2 h, 85 °C 5 min, then 4 °C (Applied Biosystems, ABI, Foster City. CA, USA, High-Capacity cDNA Reverse Transcription kit with RNAse inhibitor 4374966). Taqman probes for *Mgp* (Mm00485009_m1 to exons 1–2 boundaries), *Myoc* (Mn00447900_m1) and 18S (Hs99999901_s1) were purchased from the ABI TaqMan Gene Expression Assays. Reactions were performed in triplicate 20 µl aliquots using TaqMan Fast Universal PCR Master Mix (2 ×), no AmpErase UNG (ABI) run on a StepOnePlus Real-Time PCR System and analyzed by StepOne v.2.0.4 software (ABI). Relative Quantification values between treated and untreated samples were calculated by the formula $2^{-\Delta\Delta {\text{C}}_{\text{T}}}$ where C_T_ is the cycle at threshold, ΔC_T_ is C_T_ of the assayed gene minus C_T_ of the endogenous control (18S), and ΔΔC_T_ is the ΔC_T_ of the normalized assayed gene in the treated sample minus the ΔC_T_ of the same gene in the untreated one (calibrator). Because of the high abundance of the 18S rRNA used as the endogenous control and in order to get a linear amplification, RT reactions from treated and untreated samples were diluted 10^4^ times prior to their hybridization to the 18S TaqMan probe. Statistical analysis was performed by the Student’s t-test.

### DNA extraction and PCR analysis of the validation experiments

Genomic DNA from MIA cells was purified using a DNeasy Blood and Tissue kit (Qiagen 69054) from 35 mm dishes. Harvesting of the cells was conducted by scraping the PBS-washed 35 mm dish with a cell lifter and centrifugation at 300**×g** for 5 min. The pellet was resuspended in 200 µl PBS, 200 µl of AL buffer, 20 µl proteinase K and extraction continued following manufacturer’s recommendations. Final columns were eluted with 50 µl of HyPure water (HyClone/GE Healthcare Life Science, Logan, UT, USA). For the tissue, after PBS washing, iridocorneal strips from single eyes were disrupted by vortexing with 180 µl of the kit’s ATL plus 20 µl proteinase K. The homogenate was transferred to an eppendorf tube and extraction continued as described for the cells. In addition to iridocorneal strips, the iris and cornea tissues were also dissected and pooled each from 3 eyes. Cornea samples were homogenized in a glass microtissue grinder (Kimble-Kontes, Vineland, NJ, USA) with 180 µl of ATL plus 20 µl proteinase K prior to transferring to the eppendorf tube.

For the PCR, 2.5 µl of the DNA extracts were added to a mix containing 12.5 µl AccuStart II PCR SuperMix (Quantabio, Beverly, MA, USA), 6 µl of 2 µM each of corresponding primers, and 4 µl nuclease-free water (total 25 µl). PCR amplification conditions were 94 °C 2 min (94 °C 45 s, 65 °C 40 s, 72 °C 1 min) for 35 cycles, and ending at 72 °C for 2 min before holding the temperature at 4 °C. Primers designed to identify the *Mgp* DNA inside and outside of the floxed region were: for the inside floxed region, primer pair P14f/P14r: 5′ GTAATGTCAACCGAGGAGGCACAG 3′ and 5′ GTGACTTCAGTGGCTCACTTCAGG 3′, which yields a 661 bp amplimer of recombined *Mgp*.DNA and 2,804 bp of unrecombined DNA. For internal control outside the floxed region, we used primer pair P15f/P15r: 5′ CAACCTTGCTAAATGCACCC 3′ and 5′ GTGGCTCATGTGATGTCAGCTTAAC 3′, which yields a 392 bp amplimer. At the end of the reactions, samples were loaded onto 1% agarose (Bio-Rad, Hercules, CA, USA)/0.4 µg/ml ethidium bromide (Invitrogen/ ThermoFisher)/TBE buffer (Sigma) using a HyperLadder 1 kb DNA marker (Bioline, Thomas Scientific, Swedesboro, NJ, USA).

### Protein extraction and western blot analysis

After the removal of the medium, treated and untreated primary MIA cells in 3 cc wells were washed twice with cold PBS, and harvested in 140 µl cold lysis buffer (100 µl RIPA buffer plus 40 µl of 1** × **protease inhibitor) (Sigma-Aldrich and Roche/Sigma respectively). Lysed cells were centrifuged cold at 14,000×*g* for 10 min and the supernatant disrupted with a sonicator (Microson Ultrasonic XL 2000; Misonix, Farmingdale, NY, USA) equipped with a 2.4 mm microprobe (Misonix) at setting 3 for five pulses. The sonicate (soluble fraction) was collected and stored at − 80 °C until use. For the tissue, dissected iridocorneal angle strips, containing the trabecular meshwork, were rinsed in PBS, pooled from 3 eyes and placed in a glass microtissue grinder (Kimble-Kontes). Tissue was homogenized in steps to a total of 140 µl RIPA plus inhibitors solution as above, passed to an eppendorf tube and protein extraction continued as described for the MIA cells. In addition to iridocorneal strips, iris and cornea tissues were also dissected out and pooled each from 10 eyes. Proteins then were extracted as above.

Before electrophoresis, protein extracts were sonicated 1:2 (vol/vol) with loading Laemmli buffer (Bio-Rad) containing 5% β-mercaptoethanol and boiled for 5 min. Protein extracts were separated on 4–15% SDS-PAGE ready gels (Bio-Rad) along with Precision Plus Protein Dual Color Standards (Bio-Rad 10–250 kDa, 161-0374). After running, gels were electrotransferred to a polyvinylidene fluoride (PVDF) membrane using a Trans-Blot Turbo Transfer System (Bio-Rad). Membranes were blocked with 5% nonfat dry milk in 0.01 M Tris–HCl pH 8.0/0.2% Tween 20 for 2 h, followed by incubation overnight at 4 °C with a rabbit anti-human polyclonal MGP (1:1000 ProteinTech, Rosemont, IL, USA 10734-1) antibody. After several washes, membranes were then incubated with an HRP-conjugated goat anti-rabbit IgG antibody (1:8000, Pierce Biotechnology, Rockford, IL 31460) for 2 h. Immunoreactive bands were visualized by chemiluminescence using a SuperSignal West Femto substrate kit (ThermoFisher 34094) and developed in an Amersham Imager 600 (GE Healthcare Bio-Sciences, Pittsburgh, PA, USA). To re-probe membranes with other primary antibodies, membranes were stripped in 0.01 M Tris/ 0.1% Tween, pH 2.0, for 15 min, washed and neutralized with the same buffer at pH 8.0. For controls, membranes were incubated with a monoclonal anti-β-actin antibody for 1 h at room temperature (1:5000, Sigma A5441), washed and incubated with HRP-conjugated goat anti-mouse IgG (1:8000, Pierce 31430) for 1 h at room temperature.

### Histology

Mice were euthanized as indicated above. Whole globes were enucleated, rinsed in PBS, and immersed in fresh cold 4% paraformaldehyde (Electron Microscopy Science, EMS, Hatfield, PA. USA 19208) in PBS for 1 h at 4 °C, with a slit made a few mm below the limbus. Anterior segments were then dissected, immersed subsequently in 10% and 30% sucrose/PBS at 4 °C (about 6 h each) and embedded in OCT compound (Tissue-Tek, Sakura Finetek, Torrance, CA, USA). Cryoembedded blocks were sectioned meridionally at 10 µm thick and mounted on glass slides with DAPI-containing Fluoro-Gel II (EMS). Images were captured on an IX71 Olympus fluorescence microscope as indicated above.

### Measurement of intraocular pressures (IOP)

IOPs were measured unmasked. Mice were lightly anesthetized (42 mg/kg ketamine, 4 mg/kg xylazine and 0.8 mg/kg acepromazine) by IP injection and with an eye drop of 0.5% tetracaine (Covetrus). Measurements were obtained with a calibrated TonoLab selected for mouse settings (Colonial Medical Supply, Franconia, NH) and equipped with a foot pedal. At this anesthesia concentration, mice achieve recumbency in 3 to 5 min and all IOP measurements were taken at 3 min after recumbency. To take IOP measurements, whiskers were trimmed, and mice positioned with the visual axis horizontal to the TonoLab probe which was held at a distance 2–5 mm from the center of the cornea. IOPs were obtained as the average of 6 consecutive measurements by pressing the foot pedal. Only mean values with a standard deviation (expressed as percentage of the mean) less than 5% were accepted. The average of at least 3 such readings (18 measurements) was considered to be the absolute IOP for the given point. The Integral IOP (cumulative pressure received by each mouse during the entire duration of the experiment) was calculated using the Area Under the Curve (AUC) tool of the GraphPad Prism 5 software (GraphPad Software, Inc., La Jolla CA). Data were analyzed using the SigmaPlot software (Systat Software Inc., San Jose, CA, USA) and are presented as means ± SEM. All IOP measurements were taken between 11:30 am to 1:00 pm. Under these conditions, baseline values of the B6 strain were 8.0 ± 0.10 mmHg (n = 22 eyes).

## Supplementary information

Supplementary Information.
