# Supplementary material for: Generation of a Matrix Gla (Mgp) floxed mouse, followed by conditional knockout, uncovers a new Mgp function in the eye
Source: Sci Rep. 2020 Oct 29;10:18583. doi: 10.1038/s41598-020-75031-7 (PMC7596545; doi:10.1038/s41598-020-75031-7)
Supplement: Supplementary file 1 — Supplementary Information. [file 41598_2020_75031_MOESM1_ESM.docx]

Supplementary Information

for

Generation of a Matrix Gla (*Mgp*) floxed mouse, followed by conditional knockout, uncovers a new *Mgp* function in the eye

Teresa Borrás, Dale O. Cowley, Priyadarsini Asokan, Kumar Pandya

**Figure 1S**


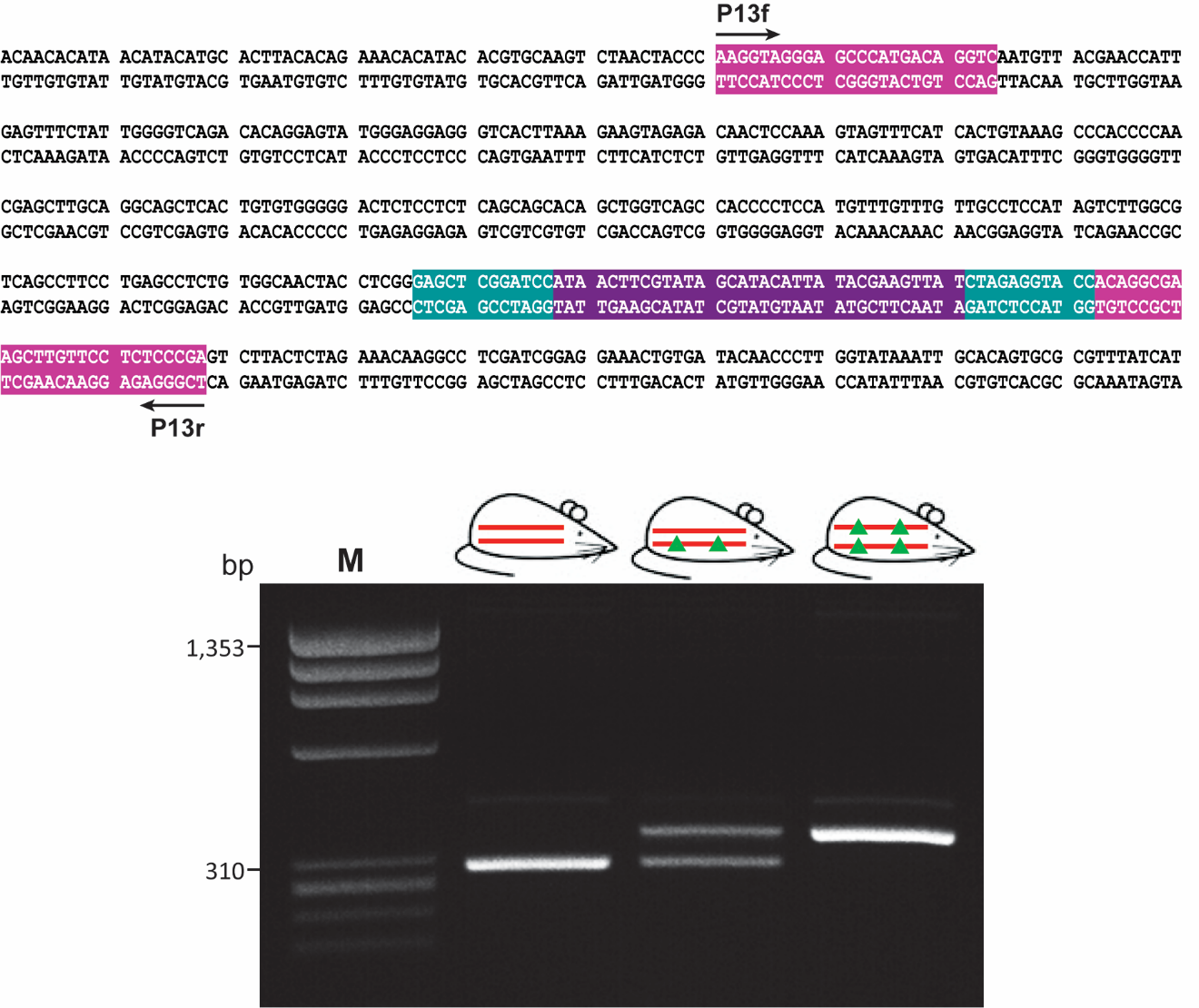


**Figure 1S.** Simplified genotyping of wild-type *Mgp^+/+^* , heterozygous *Mgp^floxed/+^* and homozygous *Mgp^floxed/floxed^* alleles. (*Top*) sequence of the *Mgp.floxed* allele surrounding the 3’loxP site*.* Purple and green: 3’loxP site sequences; pink: primer set sequences; arrows indicate name and location of the primers. (*Bottom*) Representative 2% agarose/TBE gel loaded with DNA from wild-type, heterozygous and homozygous mice. Wild-type band: 300 bp; recombined band: 358 bp. M: ΦX174 HaeIII DNA markers.

**Figure 2S**


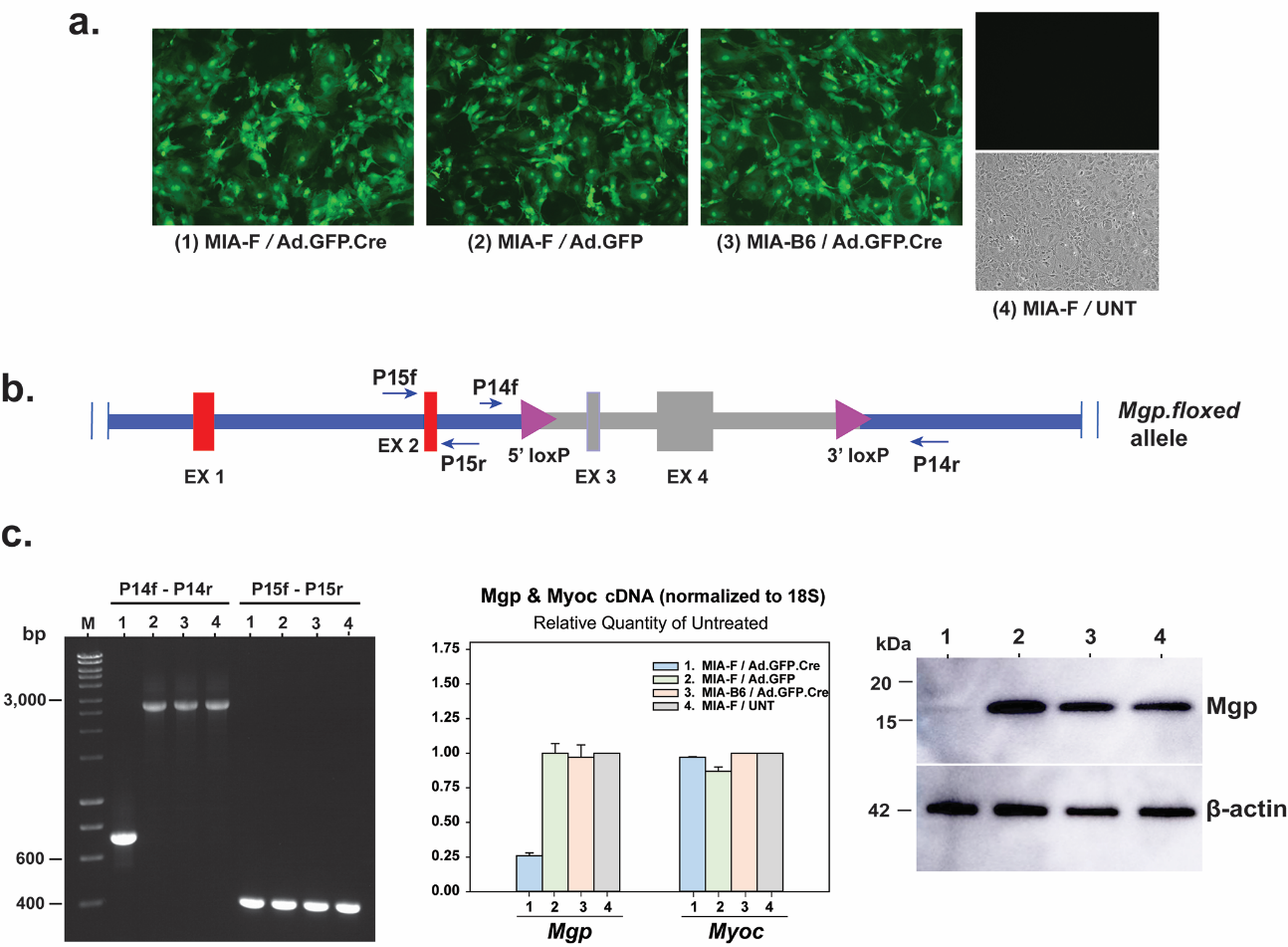


**Figure 2S.** Characterization of functional loxP sites of the *Mgp.floxed* mouse in primary mouse iridocorneal angle (MIA) cells. MIA cells from *Mgp.floxed* and B6 mice (MIA-F and MIA-B6) were infected with Ad.GFP.Cre and Ad.GFP viruses, as well as left uninfected. Group 1: MIA-F*/* Ad.GFP.Cre-infected; group 2: MIA-F*/* Ad.GFP-infected; group 3: MIA-B6/ Ad.GFP.Cre-infected; group 4: MIA-F*/* UNT uninfected. **(a)** Representative GFP green fluorescent images from MIA cells at 2 days post-infection. Transduction in all three infected groups was highly efficient. Control uninfected was negative. **(b)** Diagram of the *Mgp*.*floxed* allele. Target recombined region containing two exons is colored in gray. Arrows: primers to identify *Mgp* recombined DNA (P14f/P14r) and non-recombined control (P15f/P15r) regions. **(c)** **Left:** Representative1% agarose/TBE gel run with DNA extracted from MIA cells from the four groups (n=2-3 cells lines per group). M: HyperLadder 1kb (Bioline). The *Mgp* gene is recombined (661 bp) only in the DNA extracted from group 1 while DNA from groups 2, 3 and 4 show the 2,804 bp band of the unrecombined gene. Internal control primers yielded an equal band of 392 bp in all four groups. **(c)** **Middle**: RNA extracted from the four groups, reversed transcribed and analyzed by TaqMan PCR with *Mgp* and *Myoc* (control) probes. *Mgp* cDNA levels of group1 were 0.26±0.02 of the group 4 UNT, while the remaining groups and *Myoc* controls were not different than the UNT. **(c)** **Right:** Equivalent volumes of MIA cells protein extracts from the four groups analyzed by western blot with anti-human MGP antibody (n=3 cell lines per group). Membrane re-probed with anti-β-actin antibody as loading control. The Mgp protein is not observed in the MIA-F cells where the *Mgp* gene was recombined (group 1), while its presence is seen in the controls 2, 3 and 4. loxP sites are functional. Uncropped gels shown in Figure 4S.

**Figure 3S**


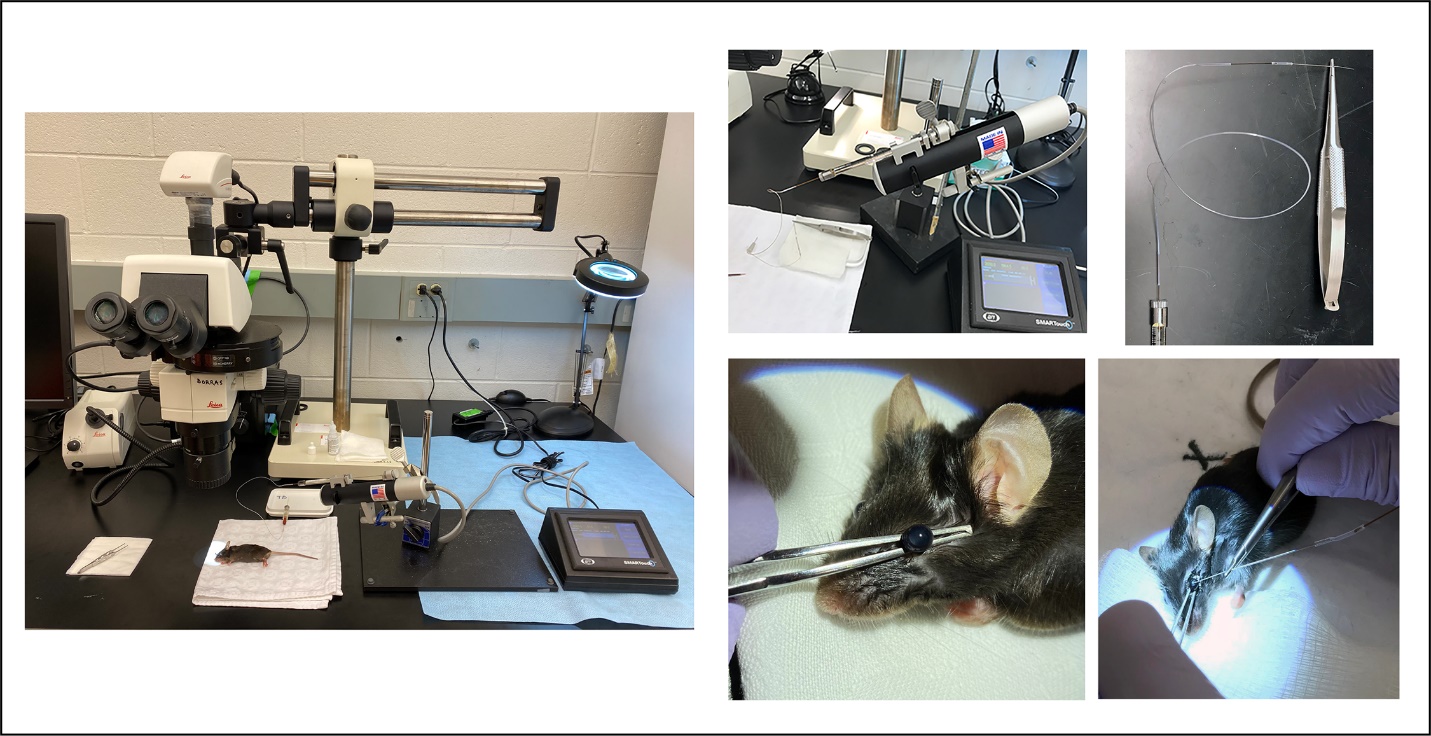


**Figure 3S.** Composite image of the mouse intracameral microinjection set up. As detailed in methods, mice are anesthetized, placed under the dissecting microscope and their eyes held by the opened area of a Bishop-Harmon forceps. NanoFil needle (33G) is especially attached to a 10 µl NanoFil syringe driven by an UltraMicroPump connected to a controller box to deliver 67 nl/s. After a gentle prick to the cornea, the nanofil needle was driven into the anterior chamber with a Barraquer needle holder. A total volume of 2 µl of experimental or control adenoviral vectors were delivered by programing the controller box and activating the pump.

**Figure 4S**


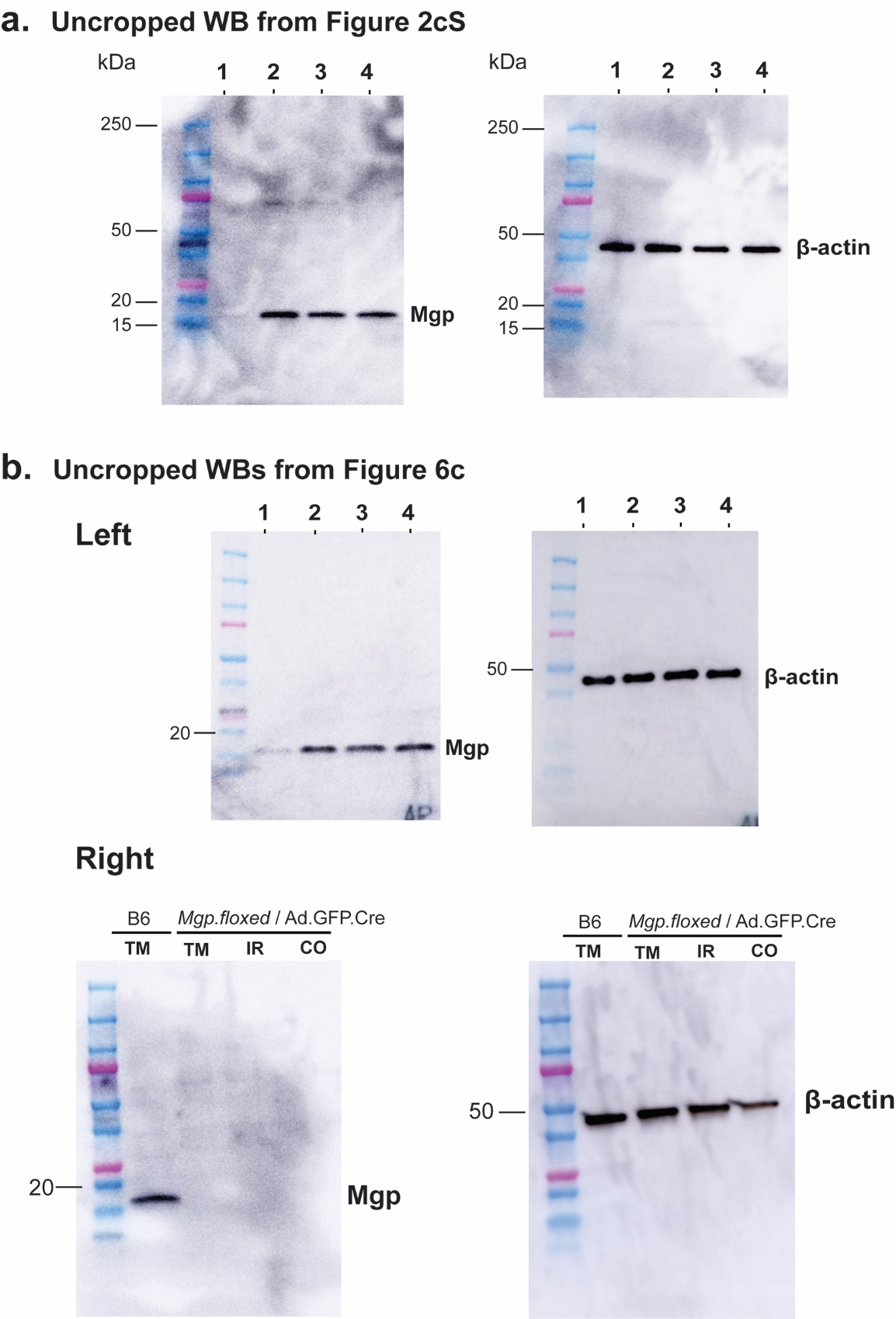


**Figure 4S.** Uncropped western blots from Figure 2cS (a) and Figure 6c (b)
